# Development and progression of hepatic steatosis during long-term glucocorticoid therapy in autoimmune hepatitis: multimodal evidence from biopsy, CAP, and ultrasound

**DOI:** 10.3389/fmed.2026.1891955

**Published:** 2026-08-05

**Authors:** Yijun Bao, Yu Zhang, Li Wang, Yongfeng Yang

**Affiliations:** 1 Nanjing University of Chinese Medicine, Nanjing, China; 2 Department of Infectious Diseases and Hepatology, the Second Hospital of Nanjing, Affiliated with Nanjing University of Chinese Medicine, Nanjing, China

**Keywords:** autoimmune hepatitis, controlled attenuation parameter, glucocorticoids, hepatic steatosis, liver biopsy, triglycerides

## Abstract

**Introduction:**

Autoimmune hepatitis (AIH) is a chronic inflammatory liver disease treated with glucocorticoids (GCs). Long-term GCs therapy may increase the risk of hepatic steatosis. This study aimed to evaluate the impact of prolonged GCs therapy on hepatic steatosis in patients with AIH.

**Methods:**

We retrospectively analyzed patients with AIH treated at a single center from January 2017 to December 2023. Eligible patients had received systemic GCs therapy for at least 24 months and had at least one paired assessment of hepatic steatosis before and after treatment, including liver histology, CAP, or abdominal ultrasound.

**Results:**

Of 160 patients, 43 (26.88%) developed new-onset or progressive hepatic steatosis. CAP values increased by 24.34 dB/m, and ultrasound-detected steatosis prevalence rose from 21.28% to 42.55%. Elevated triglyceride levels at 6 months were independently associated with new-onset or progressive hepatic steatosis.

**Discussion:**

Long-term GCs therapy was associated with a measurable increase in hepatic steatosis in patients with AIH across complementary assessment modalities. Six-month triglyceride levels may serve as an early metabolic marker for identifying patients at increased risk, supporting dynamic metabolic and steatosis monitoring during long-term immunosuppressive treatment.

## Graphical Abstract

**Figure:**
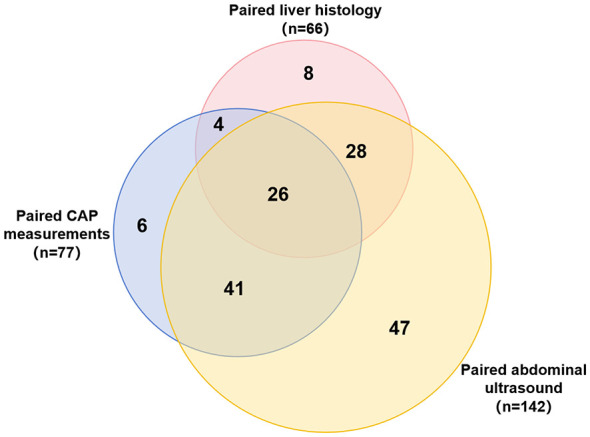
Overlap among patients with paired liver biopsy, CAP, and ultrasound data. Venn diagram showing the overlap among patients with paired liver biopsy, CAP, and ultrasound data in the final cohort (N = 160). Overall, 99 patients had paired data from more than one assessment modality, and 26 patients had paired data from all three modalities.

## Introduction

Autoimmune hepatitis (AIH) is a chronic liver disease characterized by immune-mediated hepatocellular injury. GCs remain the cornerstone of AIH treatment and have demonstrated efficacy in inducing and maintaining disease remission (1, 2). However, prolonged administration of GCs is associated with a range of metabolic side effects, such as weight gain, the development of insulin resistance, and abnormalities in lipid metabolism (3, 4).

To date, many clinical studies have explored the prevalence of nonalcoholic fatty liver disease (NAFLD) in patients with AIH, as well as the potential influence of hepatic steatosis on therapeutic efficacy and long-term clinical outcomes (5–7).

Nevertheless, the new-onset or progressive hepatic steatosis following GCs therapy in AIH patients may have important implications for disease progression, treatment efficacy, and liver-related outcomes. Relevant epidemiological data, predictive factors, and mechanistic insights remain largely unexplored.

Liver biopsy remains the gold standard for assessing hepatic steatosis. However, noninvasive methods such as the controlled attenuation parameter (CAP) measured by transient elastography and conventional abdominal ultrasound are more widely used in the evaluation of fatty liver disease. To date, there is a lack of systematic comparative studies evaluating these diagnostic modalities in patients with AIH, particularly in the context of GCs-associated metabolic alterations.

Our study aimed to evaluate the development and progression of hepatic steatosis in patients with AIH following GCs therapy using a multimodal approach, including liver biopsy, CAP measurement, and abdominal ultrasound (8, 9). Furthermore, we sought to identify clinical and biochemical indicators associated with steatosis progression in order to provide insight into metabolic monitoring and risk stratification in this specific patient population.

## Methods

### Patients

We performed a retrospective longitudinal analysis of patients diagnosed with AIH at the Second Hospital of Nanjing between January 2017 and December 2023. AIH was diagnosed according to the revised 1999 criteria of the International Autoimmune Hepatitis Group (IAIHG), and liver histology at diagnosis was available for all included patients. During follow-up, patients did not receive any structured dietary or exercise interventions as part of routine clinical management.

Exclusion criteria were as follows: (1) overlap with primary biliary cholangitis, primary sclerosing cholangitis, IgG4-related sclerosing cholangitis, or other chronic liver diseases; (2) discontinuous glucocorticoid therapy or glucocorticoid treatment for less than 24 months; (3) treatment with budesonide; (4) long-term systemic GCs therapy for diseases other than AIH; (5) age < 18 years; (6) a history of heavy alcohol consumption during the treatment period; (7) absence of paired hepatic steatosis assessment before and after glucocorticoid therapy; or (8) incomplete or missing key clinical data.

Based on these criteria, 232 patients with pure AIH were initially identified. Among them, 160 patients with complete paired liver histology, CAP, or ultrasound data before and after treatment were included in the final analysis.

This study was approved by the Ethics Committee of the Second Hospital of Nanjing (2022-LY-kt101). Given the retrospective nature of the study and the use of de-identified clinical data, the requirement for written informed consent was waived by the Ethics Committee. All procedures were conducted in accordance with the Declaration of Helsinki.

### Treatment and outcomes

Patients initially received oral methylprednisolone therapy at a daily dose ranging from 8 to 40 mg. The dose was gradually tapered. For patients receiving other glucocorticoids, doses were converted to methylprednisolone equivalents. Azathioprine or mycophenolate mofetil was subsequently added for combination therapy.

Complete biochemical remission was defined as the normalization of serum aminotransferases and immunoglobulin G (IgG) levels within 6 months. Incomplete biochemical remission was defined as partial improvement in laboratory parameters within 6 months, with serum aspartate aminotransferase (AST), alanine aminotransferase (ALT), or IgG levels remaining above the normal range.

### Liver histology

Liver histological data at the time of diagnosis were available for all patients. A total of 66 individuals underwent two or more histopathological assessments during follow-up. All histological examinations and scoring were independently performed by two experienced liver pathologists on the same day in a blinded manner, without access to clinical information. The severity of hepatic inflammation was assessed using the histological activity index (HAI), with a total score ranging from 0 to 18. Histological remission was defined as normal liver histology or mild hepatitis (HAI ≤ 3), while persistent histological activity was defined as HAI ≥ 4 (10).

### CAP measurement and abdominal ultrasound

As part of routine clinical care, patients underwent CAP measurement and abdominal ultrasound examinations. Both procedures were performed by experienced radiologists. Given the retrospective design of this study, it was not possible to confirm whether the operators performing the CAP or the radiologists conducting the ultrasound were blinded to the patients' clinical data or other test results at the time of the examinations; furthermore, the pre- and post-treatment assessments were not necessarily performed by the same operator.

### New-onset or progression of hepatic steatosis

For patients with paired liver biopsy data, the percentage of hepatocellular steatosis was assessed on both baseline and follow-up specimens. The difference in steatosis percentage between the two time points was calculated to evaluate the change. Histologically defined new-onset or progressive hepatic steatosis was determined based on the following criteria: (1) Progression: hepatic steatosis was present at diagnosis, with an increase of ≥20% in the steatotic area on follow-up biopsy; (2) New-onset: no hepatic steatosis was present at diagnosis, but steatosis was detected on the second histopathological examination. All remaining patients were classified as having no histologically defined progression.

Abdominal ultrasound was performed by experienced radiologists using routine clinical protocols. Ultrasound-defined hepatic steatosis was diagnosed based on increased hepatic echogenicity relative to the renal cortex, attenuation of the ultrasound beam, and reduced visualization of intrahepatic vascular structures. For the present analysis, ultrasound findings were dichotomized as steatosis present or absent.

For patients with complete CAP data before and after treatment, hepatic steatosis was defined as a CAP value ≥ 248 dB/m (11, 12). CAP-defined new-onset steatosis was defined as baseline CAP < 248 dB/m and follow-up CAP ≥ 248 dB/m. Among patients with baseline CAP ≥ 248 dB/m, CAP-defined progression was defined as an increase in CAP of ≥ 20 dB/m during follow-up.

According to the criteria proposed by Sasso (13), we used CAP ≥ 248 dB/m as the diagnostic threshold for hepatic steatosis. Studies such as that by Bai et al. (14) have also shown that the optimal CAP threshold for distinguishing normal liver fat content from steatosis is approximately 247 dB/m. Given its higher sensitivity, this threshold may be more suitable for detecting mild hepatic steatosis. However, more recent studies and guidelines, including Eddowes et al. (15) and the EASL 2021 guideline (16), suggest that higher CAP thresholds, such as 274 dB/m, provide greater specificity and may be more appropriate for identifying more advanced steatosis. We chose 248 dB/m as the cut-off for the primary analysis because of its higher sensitivity for detecting early hepatic steatosis in patients with AIH. Nevertheless, we also performed a sensitivity analysis using 274 dB/m as a more conservative cut-off.

### Hierarchical assignment for the overall cohort analysis

Patients in the liver biopsy cohort were required to have at least two histopathological assessments, with the first biopsy performed before December 2023 and an interval of at least 24 months between biopsies. Patients in the CAP and ultrasound cohorts were required to have both baseline assessments before GCs initiation and follow-up data obtained after more than 24 months of GCs therapy.

For the overall cohort analysis, the primary endpoint was new-onset or progressive hepatic steatosis after at least 24 months of GCs therapy. Because hepatic steatosis was assessed using different modalities in routine practice, a hierarchical composite endpoint was adopted. When multiple modalities were available for the same patient, hepatic steatosis status was determined according to the following predefined priority order: liver histology, followed by CAP, and then abdominal ultrasound. Patients were assigned to the liver biopsy cohort if paired histological assessments before and after treatment were available. Patients without paired biopsy data but with paired CAP measurements were assigned to the CAP cohort. Patients with neither paired biopsy nor paired CAP data, but with paired abdominal ultrasound examinations, were assigned to the ultrasound cohort. Each patient was included only once in the overall cohort analysis according to the highest-priority modality available.

### Statistical analysis

Data organization and statistical analyses were performed using R software, version 4.3.3 and 4.4.1. Normally distributed continuous variables are presented as mean ± standard deviation and were compared using Student's *t*-test. Non-normally distributed continuous variables are presented as median and interquartile range and were compared using the Mann–Whitney *U*-test. Categorical variables are presented as counts and percentages and were compared using the chi-square test or Fisher's exact test, as appropriate. Paired categorical changes in hepatic steatosis status before and after glucocorticoid therapy were evaluated using McNemar's test. Paired continuous variables, including CAP values and metabolic parameters at different time points, were compared using paired *t*-tests or Wilcoxon signed-rank tests, according to data distribution. The primary outcome was new-onset or progressive hepatic steatosis after at least 24 months of glucocorticoid therapy, defined using the hierarchical assessment order of liver histology, CAP, and abdominal ultrasound. Univariable logistic regression was first used to explore factors associated with this outcome. Multivariable logistic regression models were then constructed using clinically relevant variables and variables showing potential associations in univariable analyses. Given the limited number of outcome events, the number of covariates was restricted to reduce the risk of overfitting. Subgroup regression analyses were considered exploratory. Odds ratios (ORs) and 95% confidence intervals (CIs) were reported. Receiver operating characteristic (ROC) curve analysis was used to evaluate the predictive performance of 6-month triglyceride levels for new-onset or progressive hepatic steatosis. The optimal cut-off value was determined using the Youden index. Cohen's kappa coefficients were calculated to assess pairwise agreement among liver histology, CAP, and ultrasound. Liver histology was used as the reference standard for calculating the sensitivity and specificity of CAP and ultrasound. A sensitivity analysis was performed using a CAP threshold of 274 dB/m, in addition to the primary threshold of 248 dB/m. All statistical tests were two-sided, and *p* < 0.05 was considered statistically significant. Exploratory interaction analyses were performed by adding multiplicative interaction terms to the logistic regression model. Continuous variables were mean-centered before constructing interaction terms.

## Results

## Clinical characteristics of entire AIH cohort

**Figure 1:**
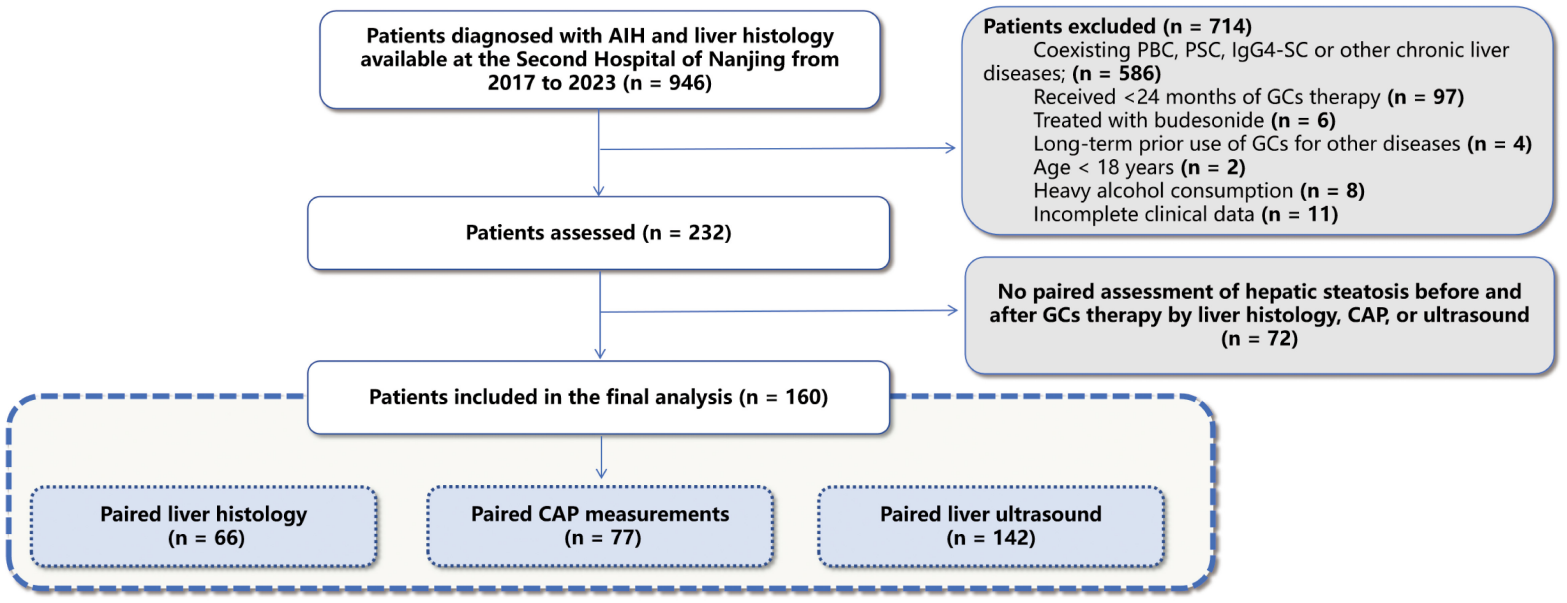
Flowchart of patient selection and subgroup classification. A total of 946 patients diagnosed with autoimmune hepatitis (AIH) with liver histology available at the Second Hospital of Nanjing between 2017 and 2023 were screened. After excluding patients with coexisting PBC, PSC, IgG4-SC, or other chronic liver diseases (*n* = 586), those who received < 24 months of GCs therapy (*n* = 97), were treated with budesonide (*n* = 6), had long-term prior use of GCs for other diseases (*n* = 4), were younger than 18 years (*n* = 2), had heavy alcohol consumption (*n* = 8), or had incomplete clinical data (*n* = 11), 232 patients were assessed. An additional 72 patients were excluded because paired assessment of hepatic steatosis before and after GCs therapy by liver histology, CAP, or ultrasound was unavailable. Ultimately, 160 patients were included in the final analysis. Among these patients, paired liver histology, paired CAP measurements, and paired abdominal ultrasound data were available in 66, 77, and 142 patients, respectively. For the hierarchical overall analysis, patients were assigned to the biopsy group (*n* = 66), CAP group (*n* = 47), or ultrasound group (*n* = 47).

A total of 160 patients with AIH were included in the study (Figure 1). Among them, 66 patients had paired liver histopathology data, 77 had CAP measurements before and after treatment, and 142 had pre- and post-treatment abdominal ultrasound data. Overlaps existed among the subgroups (Supplementary Figure S1). Patients were hierarchically assigned to the liver biopsy group, followed by the CAP group, and then the ultrasound group. If a patient did not undergo a second liver biopsy and lacked paired CAP measurements, they were assigned to the ultrasound group.

66 patients were included in the liver biopsy group, 47 in the CAP group, and 47 in the ultrasound group. The clinical characteristics of each subgroup are summarized in Supplementary Table S1. Overall, baseline differences among the three groups were minimal. Patients in the liver biopsy group had higher baseline ALB and lower ALT, AST, GGT, and IgG levels compared to the other groups, but these differences were directionally consistent. No statistically significant differences were observed in other clinical parameters among the groups.

In the overall AIH cohort, 43 patients (26.88%) developed new-onset or progressive hepatic steatosis after receiving GCs treatment for more than 24 months, while 117 patients (73.12%) exhibited stable or regressive steatosis. To identify factors associated with steatosis progression, the clinical characteristics of these patients were systematically reviewed (Table 1).

**Table 1:** Clinical characteristics of the overall cohort.

Variables	Newly Developed or Progressive Hepatic Steatosis (*n* = 43)	Stable, Regressed, or Without Hepatic Steatosis (*n* = 117)	*p*
Female (%)	38 (88.37)	91 (77.78)	0.133
Age at disease onset (years)	51.00 (44.50, 59.00)	53.00 (46.00, 60.00)	0.648
BMI	23.63 (22.08, 25.57)	22.66 (20.70, 24.46)	0.052
Initial dose of glucocorticoids (mg)	24.00 (24.00, 32.00)	24.00 (16.00, 32.00)	0.037
CBR at 6 month (%)	26 (60.47)	68 (58.12)	0.789
ANA (%)	28 (65.12)	85 (72.65)	0.354
SMA (%)	4 (9.30)	11 (9.40)	1.000
Laboratory test at diagnosis			
RBC ( × 10^12^/L)	4.25 ± 0.55	3.93 ± 0.56	0.002
Hb (g/L)	129.37 ± 14.53	123.32 ± 18.54	0.055
WBC ( × 10^9^/L)	5.32 (4.26, 9.38)	4.53 (3.56, 7.74)	0.127
PLT ( × 10^9^/L)	149.00 (121.50, 191.00)	127.00 (95.00, 174.00)	0.037
ALB (g/L)	39.37 ± 4.17	37.30 ± 5.16	0.019
GLO (g/L)	30.00 (26.55, 36.65)	28.90 (25.60, 32.70)	0.134
ALT (U/L)	180.20 (53.55, 357.25)	92.70 (47.70, 201.60)	0.064
AST (U/L)	175.80 (55.80, 290.75)	111.30 (52.50, 275.50)	0.525
γ-GT (U/L)	131.00 (59.45, 205.10)	92.00 (46.50, 169.40)	0.249
ALP (U/L)	103.00 (81.25, 140.00)	117.20 (86.00, 161.00)	0.215
IgG (g/L)	16.40 (12.95, 18.50)	16.10 (12.10, 20.50)	0.876
FBG (mmol/L)	5.01 (4.35, 5.48)	4.72 (4.38, 5.29)	0.274
Cholesterol (mmol/L)	3.72 (3.26, 4.37)	3.77 (3.06, 4.79)	0.695
Triglycerides (mmol/L)	1.40 (1.08, 2.19)	1.24 (0.89, 1.80)	0.217
High-density lipoprotein (mmol/L)	1.08 (0.79, 1.52)	1.11 (0.77, 1.74)	0.343
Low-density lipoprotein (mmol/L)	2.01 ± 0.85	2.06 ± 0.82	0.736
Laboratory test after 6-month immunosuppressive therapy			
ALT (U/L)	23.00 (18.05, 29.97)	26.50 (18.10, 44.70)	0.171
AST (U/L)	25.40 (19.50, 31.15)	27.20 (21.10, 39.98)	0.162
γ-GT (U/L)	26.40 (18.00, 38.45)	28.00 (15.20, 56.00)	0.599
ALP (U/L)	60.00 (49.45, 77.95)	68.00 (55.00, 87.00)	0.047
FBG (mmol/L)	5.50 (4.94, 6.07)	5.07 (4.71, 5.65)	0.036
Cholesterol (mmol/L)	5.68 (4.78, 6.50)	4.90 (4.07, 5.50)	< 0.001
Triglycerides (mmol/L)	1.87 (1.25, 3.16)	1.10 (0.85, 1.62)	< 0.001
High-density lipoprotein (mmol/L)	1.58 (1.31, 1.93)	1.62 (1.31, 2.06)	0.761
Low-density lipoprotein (mmol/L)	3.50 (2.52, 4.47)	2.70 (2.11, 3.24)	0.004
IgG (g/L)	12.70 (10.30, 14.80)	12.00 (9.80, 14.35)	0.710

At the time of diagnosis, patients with new-onset or progressive hepatic steatosis had significantly higher red blood cell counts (RBC: 4.25 ± 0.55 vs. 3.93 ± 0.56 × 10^1^^2^/L, *p* = 0.002), platelet counts (PLT: 149 vs. 127 × 10^9^/L, *p* = 0.037), and serum ALB levels (39.37 ± 4.17 vs. 37.30 ± 5.16 g/L, *p* = 0.019) compared with those with stable, regressive, or no hepatic steatosis.

After 6 months of GCs therapy, patients with steatosis progression had lower alkaline phosphatase levels (ALP: 60 vs. 68 U/L; *p* = 0.047) and more pronounced disturbances in glucose and lipid metabolism. Fasting blood glucose (FBG: 5.5 vs. 5.07 mmol/L, *p* = 0.036), total cholesterol (TC: 5.68 vs. 4.90 mmol/L, *p* < 0.001), TG (1.87 vs. 1.10 mmol/L, *p* < 0.001), and low-density lipoprotein cholesterol (LDL-C: 3.5 vs. 2.7 mmol/L, *p* = 0.004) were significantly higher in the progression group.

## Histological progression of hepatic steatosis

**Figure 2:**
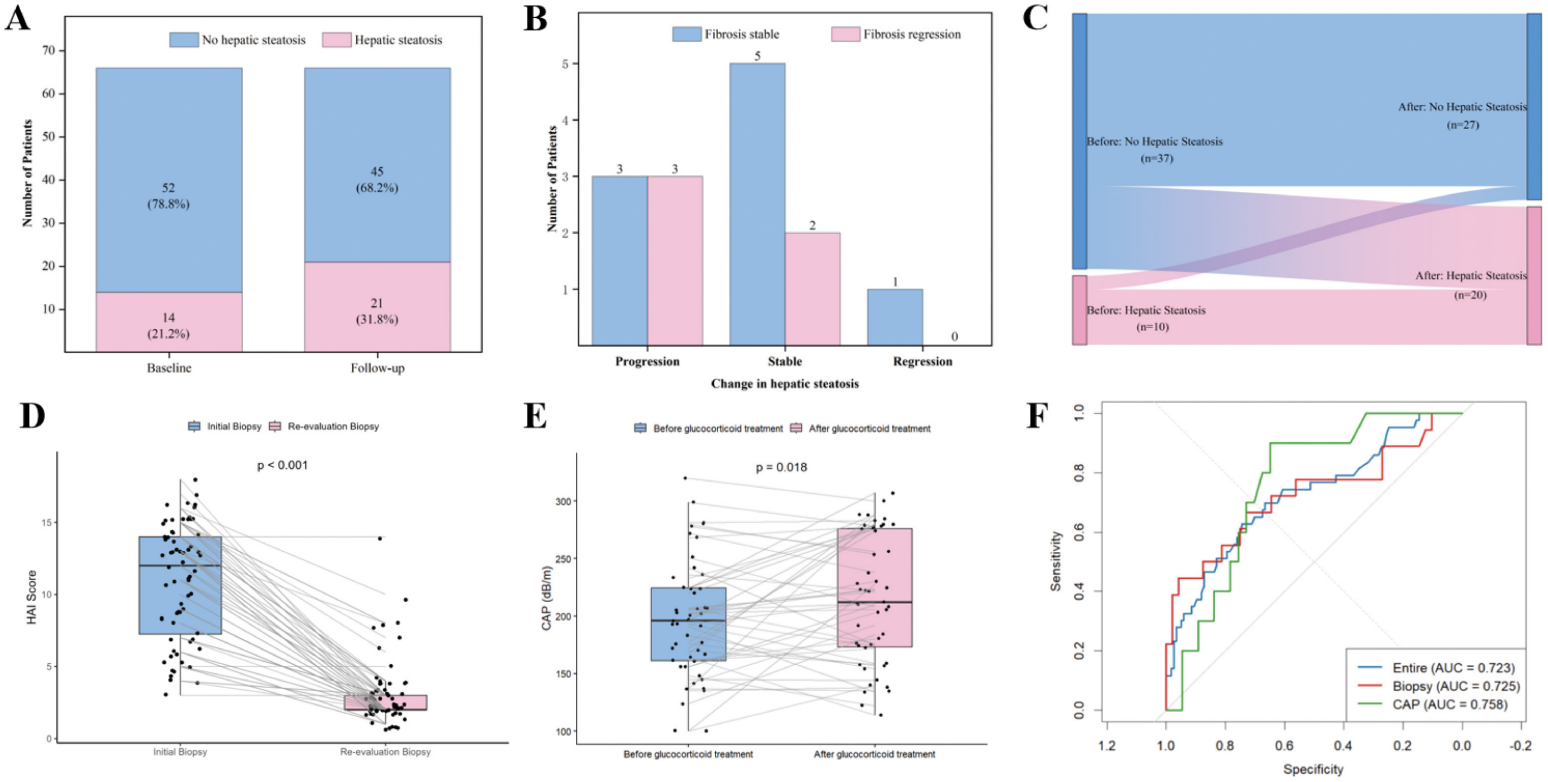
Multimodal evaluation of steatosis progression and its predictors in AIH patients during glucocorticoid therapy. **(A)** Distribution changes of hepatic steatosis status in 66 AIH patients based on initial and follow-up liver biopsies. Bars are divided into Pure AIH and AIH with steatosis, illustrating the progression of steatosis during follow-up. **(B)** Distribution of fibrosis trends according to hepatic steatosis dynamics in patients with serial liver biopsies. **(C)** Sankey diagram showing changes in ultrasound-detected hepatic steatosis before and after glucocorticoid therapy. **(D)** Paired changes in histological activity index (HAI) between the initial and follow-up liver biopsies, showing a significant decrease during follow-up. **(E)** Paired changes in controlled attenuation parameter (CAP) before and after glucocorticoid therapy, showing a significant increase after treatment. **(F)** Receiver operating characteristic (ROC) curves showing the predictive performance of 6-month triglyceride levels for new-onset or progressive hepatic steatosis in the overall cohort, biopsy cohort, and CAP cohort.

We analyzed the clinical data of 66 patients who underwent paired liver biopsies. At the time of the first histological assessment, hepatic steatosis was present in 14 patients (21.21%), whereas 52 patients (78.79%) showed no evidence of steatosis. At the second histological evaluation, 21 patients (31.82%) had histological evidence of hepatic steatosis, whereas 45 patients (68.18%) did not (Figure 2A). Notably, 8 patients (12.12%) had steatosis on both the first and second biopsies.

Based on changes in steatosis status, the study population was stratified into two groups. A total of 18 patients (27.17%) showed new-onset hepatic steatosis or histological progression of steatosis at the second biopsy, while 48 patients (72.73%) had stable, regressive, or no hepatic steatosis throughout the follow-up. The clinical and laboratory characteristics of the two groups are presented in Table 2.

**Table 2:** Clinical characteristics of AIH patients with twice liver histopathological examination (*n* = 66).

Variable	Newly Developed or Progressive Hepatic Steatosis (*n* = 18)	Stable, Regressed, or Without Hepatic Steatosis (*n* = 48)	*p* value
Female (%)	15 (83.33%)	38 (79.17%)	0.705
Age at disease onset (years)	54 (48, 60.25)	53 (44.5, 60)	0.387
Initial dose of glucocorticoids (mg)	24 (24, 32)	24 (18, 32)	0.293
CBR at 6 month (%)	13 (72.22%)	27 (56.25%)	0.237
ANA (%)	12 (66.67%)	36 (75%)	0.498
SMA (%)	3 (16.67%)	5 (10.42%)	0.488
Laboratory test at diagnosis			
RBC ( × 10^12^/L)	4.46 ± 0.43	3.98 ± 0.51	0.001
Hb (g/L)	136.82 ± 10.75	123.69 ± 17.89	0.005
WBC ( × 10^9^/L)	5.92 (4.36, 10.29)	4.44 (3.53, 7.59)	0.027
PLT ( × 10^9^/L)	144.34 ± 51.51	148.56 ± 67.71	0.812
ALB (g/L)	40.83 ± 4.37	38.41 ± 4.29	0.047
GLO (g/L)	31.69 ± 7.64	27.89 ± 5.67	0.032
ALT (U/L)	54.5 (17.72, 343.02)	63.3 (16.45, 281.17)	0.751
AST (U/L)	49.95 (22.97, 254.4)	76.25 (24.5, 164.37)	0.526
γ-GT (U/L)	87 (25.5, 142.47)	70.1 (20.7, 123.32)	0.768
ALP (U/L)	93 (70, 134.65)	103 (71.5, 164.15)	0.38
IgG (g/L)	16.35 (11.75, 17.75)	13.6 (11.32, 18.42)	0.512
Laboratory test after 6-month immunosuppressive therapy			
ALT (U/L)	22.75 (17.97, 25.5)	24.5 (16.07, 38.65)	0.357
AST (U/L)	25.6 (17.77, 30.07)	27.15 (20.9, 38.35)	0.075
γ-GT (U/L)	22.9 (14.75, 35.47)	21.4 (14.85, 54.62)	0.903
ALP (U/L)	57.9 (49.75, 81.84)	64.8 (51.25, 86.45)	0.517
IgG (g/L)	11.97 ± 3.2	12.2 ± 3.91	0.825

Patients with new-onset or progressive steatosis at the second biopsy had significantly higher baseline levels of RBC (4.47 ± 0.43 vs. 3.98 ± 0.51 × 10^1^^2^/L, *p* = 0.001), hemoglobin (Hb: 136.82 ± 10.75 vs. 123.69 ± 17.89 g/L, *p* = 0.005), white blood cells count (WBC: 5.92 vs. 4.44 × 10^9^/L, *p* = 0.027), ALB (40.83 vs. 38.41 g/L, *p* = 0.047), and globulin (GLO: 31.69 vs. 27.89 g/L, *p* = 0.032) compared with those without steatosis progression.

Among the 14 patients who initially had hepatic steatosis, follow-up histological analysis showed that fibrosis remained stable in nine patients and regressed in five (Figure 2B). In total, 13 patients developed new-onset steatosis after GCs treatment, and 6 patients demonstrated histological regression of steatosis (Table 3). The corresponding proportions were 19.7% (95% CI: 10.9% to 31.3%) and 9.1% (95% CI: 3.4% to 18.7%), respectively. Although worsening was numerically more frequent than regression, the paired comparison did not reach statistical significance (McNemar *p* = 0.167). The paired net difference was 10.6% (95% CI: −2.1% to 23.3%). This suggests a possible trend toward steatosis worsening after glucocorticoid therapy, although the difference did not reach statistical significance.

**Table 3:** Paired changes in histologically defined hepatic steatosis before and after GCs therapy among patients with paired liver biopsies (*n* = 66).

Hepatic steatosis status	With Hepatic steatosis after treatment	Without hepatic steatosis after treatment
With Hepatic steatosis before treatment	8	6
Without hepatic steatosis before treatment	13	39

The proportion of patients with worsening transition was 13/66 (19.7%, 95% CI: 10.9%−31.3%), whereas the proportion with regression was 6/66 (9.1%, 95% CI: 3.4%−18.7%). The paired net difference was 10.6% (95% CI: −2.1% to 23.3%).

## Quantitative changes in steatosis on follow-up biopsies

In our cohort, only eight patients exhibited hepatic steatosis in liver biopsy specimens at both baseline and follow-up. Histological slides from these patients were re-evaluated to quantitatively assess the degree of steatosis, along with steatosis type, distribution range, and pattern (Supplementary Table S2).

The results showed that in three patients, the absolute change in steatosis percentage remained within ±20%, indicating relatively stable steatosis levels that were not markedly affected by GCs treatment. In contrast, five patients (62.5%) showed progression of steatosis. Among them, one patient exhibited significant progression, with hepatic steatosis increasing from 5% at baseline to 35% at follow-up, while the remaining four patients showed moderate progression.

**Figure 3:**
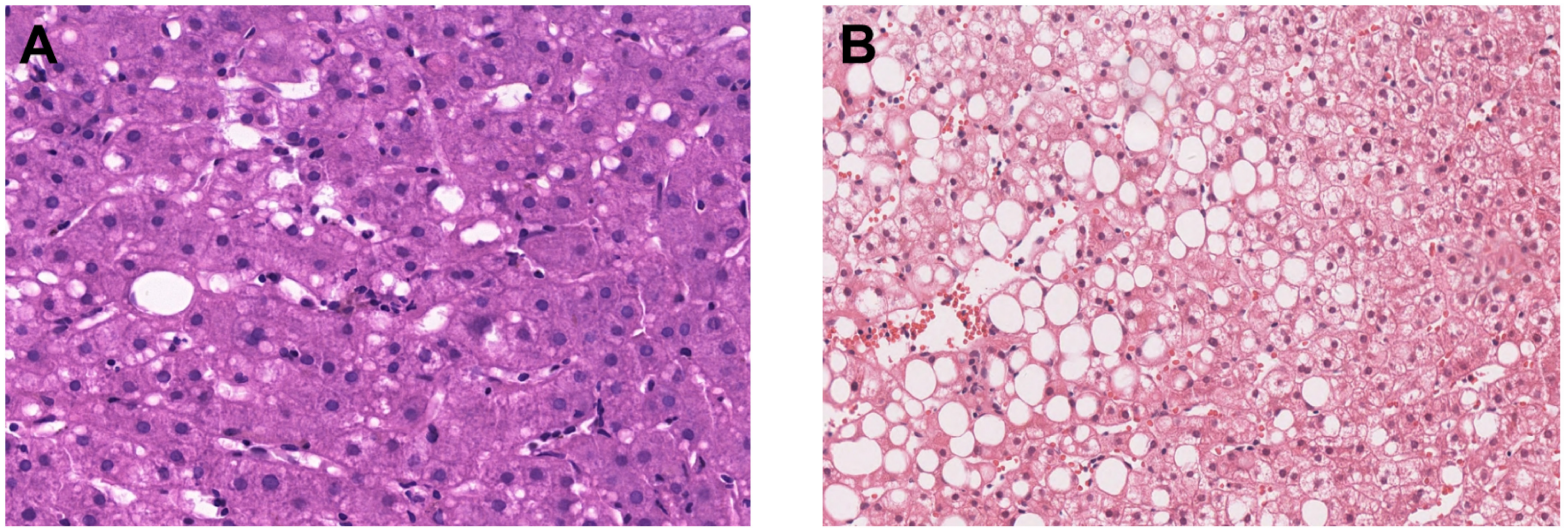
Representative paired liver biopsy sections showing progression of hepatic steatosis during long-term GCs therapy in patients with autoimmune hepatitis**. (A)** Baseline liver biopsy specimen from a patient with AIH before GCs therapy. **(B)** Follow-up liver biopsy specimen from the same patient after long-term GCs therapy, showing increased hepatocellular steatosis. Sections were stained with hematoxylin and eosin (H&E).

Before GCs treatment, 50% of the patients had a focal steatosis pattern, while the other 50% showed diffuse panlobular distribution. In terms of zonation, steatosis was predominantly located in zone three in most patients (75%). In two patients, steatotic hepatocytes were also observed in zone two and periportal (zone 1) regions. Overall, GCs therapy was associated with an increasing trend in hepatic fat content among patients with AIH and hepatic steatosis (Figure 3).

**Figure 4:**
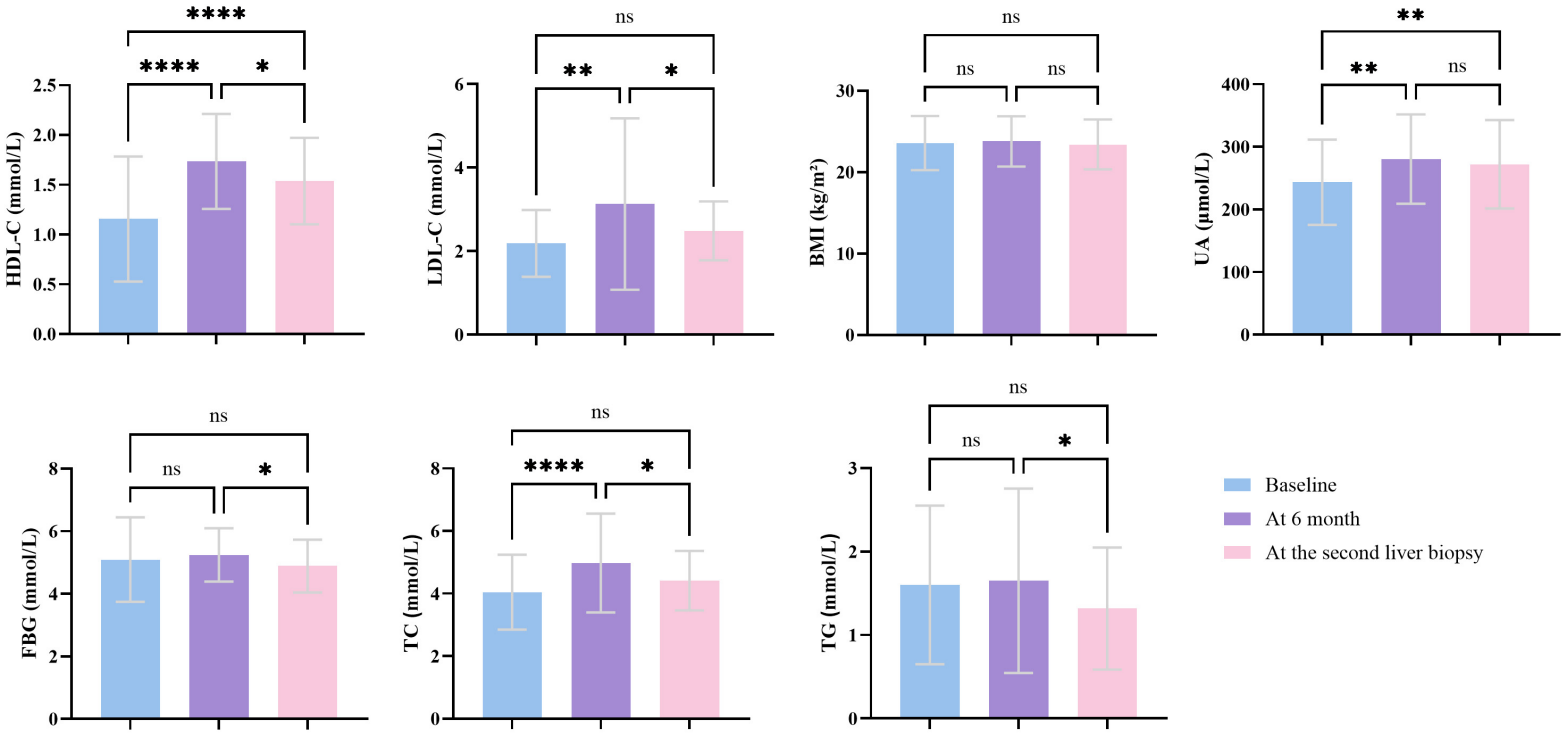
Dynamic changes in metabolic parameters in AIH patients with paired liver biopsies during glucocorticoid therapy. HDL-C, LDL-C, BMI, UA, FBG, TC, and TG were evaluated at baseline, at 6 months after glucocorticoid therapy, and at the time of the second liver biopsy in AIH patients with paired liver biopsy data. Pairwise comparisons between time points are shown above each panel. ns indicates no significant difference; *p < 0.05, **p < 0.01, and ****p < 0.0001.

HAI scores were evaluated for all 66 patients. Post-treatment scores showed a significant reduction (*p* < 0.001). A total of 51 patients (77.27%) achieved histological remission (HAI < 4). Among those with persistent histological activity (HAI ≥ 4), nine patients (13.64%) had mild activity (HAI 4–6), and only one patient had a score of 7. 40 patients (60.61%) achieved biochemical remission. These findings suggest that histological inflammatory activity in AIH patients improved significantly after GCs therapy (Figure 2D).

## Metabolic changes in patients with paired liver biopsy

To investigate whether GCs interfere with the metabolic status of AIH patients, we performed dynamic monitoring of metabolic indicators in patients who underwent secondary liver biopsy (Figure 4). After 6 months of glucocorticoid treatment, there was a significant increase in HDL-C, LDL-C, UA, and TC levels in AIH patients. Furthermore, in patients with progression of hepatic steatosis, TC (5.85 vs. 4.80 mmol/L, *p* = 0.014), TG (1.95 vs. 1.23 mmol/L, *p* = 0.005), LDL-C (3.29 vs. 2.69 mmol/L, *p* = 0.032), and UA (313.72 vs. 268.17 μmol/L, *p* = 0.020) levels were significantly higher (Table 4), compared to the other group. Additionally, patients with progressive hepatic steatosis had a significantly higher BMI at the 6-month treatment and secondary liver biopsy time points compared to the other group.

**Table 4:** Metabolic characteristics of patients with secondary liver biopsy.

Variables		Total (*n* = 66)	Newly Developed or Progressive Hepatic Steatosis (*n* = 18)	Stable, Regressed, or Without Hepatic Steatosis (*n* = 48)	Statistic	*p*
BMI	At diagnosis	23.65 (21.62, 25.32)	23.65 (22.69, 26.05)	23.68 (21.32, 24.70)	*Z* = −1.07	0.287
	At 6 month	23.85 (21.53, 25.68)	24.93 (23.65, 26.52)	23.40 (21.20, 25.31)	*Z* = −2.00	0.045
	At the second liver biopsy	23.59 (21.58, 24.94)	24.35 (23.49, 26.05)	23.14 (21.19, 24.78)	*Z* = −2.08	0.037
TG (mmol/L)	At diagnosis	1.26 (1.01, 2.10)	1.37 (1.13, 2.17)	1.22 (0.98, 1.91)	*Z* = −0.84	0.404
	At 6 month	1.42 (0.86, 1.94)	1.95 (1.41, 3.28)	1.23 (0.83, 1.65)	*Z* = −2.79	0.005
	At the second liver biopsy	1.19 (0.86, 1.43)	1.42 (1.23, 2.29)	1.10 (0.83, 1.36)	*Z* = −2.89	0.004
TC (mmol/L)	At diagnosis	4.05 ± 1.20	4.23 ± 1.27	3.98 ± 1.18	*t* = −0.77	0.444
	At 6 month	4.98 (4.13, 5.82)	5.85 (4.42, 6.69)	4.80 (4.08, 5.15)	*Z* = −2.47	0.014
	At the second liver biopsy	4.21 (3.76, 5.09)	4.71 (4.08, 5.14)	4.12 (3.74, 5.04)	*Z* = −1.38	0.169
HDL-C (mmol/L)	At diagnosis	1.16 ± 0.63	1.07 ± 0.43	1.19 ± 0.69	*t* = 0.72	0.476
	At 6 month	1.73 ± 0.48	1.69 ± 0.42	1.75 ± 0.50	*t* = 0.50	0.618
	At the second liver biopsy	1.54 ± 0.43	1.50 ± 0.52	1.55 ± 0.40	*t* = 0.39	0.694
LDL-C (mmol/L)	At diagnosis	2.19 ± 0.80	2.29 ± 0.90	2.15 ± 0.77	*t* = −0.64	0.527
	At 6 month	2.74 (2.25, 3.32)	3.29 (2.72, 4.02)	2.69 (2.17, 3.01)	*Z* = −2.13	0.032
	At the second liver biopsy	2.28 (2.02, 2.85)	2.52 (2.13, 3.04)	2.23 (1.94, 2.74)	*Z* = −0.84	0.404
FBG (mmol/L)	At diagnosis	4.76 (4.45, 5.33)	5.01 (4.54, 5.44)	4.62 (4.43, 5.11)	*Z* = −1.06	0.290
	At 6 month	5.04 (4.62, 5.64)	5.58 (4.93, 5.83)	5.01 (4.60, 5.50)	*Z* = −1.67	0.095
	At the second liver biopsy	4.81 (4.42, 5.40)	4.87 (4.29, 5.50)	4.77 (4.43, 5.22)	*Z* = −0.64	0.522
UA (μmol/L)	At diagnosis	243.45 ± 68.04	252.33 ± 54.47	240.12 ± 72.72	*t* = −0.65	0.520
	At 6 month	280.59 ± 71.35	313.72 ± 64.24	268.17 ± 70.49	*t* = −2.39	0.020
	At the second liver biopsy	272.24 ± 70.60	301.83 ± 72.67	261.15 ± 67.24	*t* = −2.14	0.036

## Association between GGT and hepatic steatosis at follow-up liver biopsy

Our study revealed that patients with hepatic steatosis at the time of the second liver biopsy had significantly higher GGT levels (19.00 vs. 15.30 U/L, *p* = 0.047) and AST levels (24.5 vs. 17.4 U/L, *p* = 0.025) compared to those without steatosis. However, no significant differences were observed in serum IgG, TC, or TG between the two groups. In addition, the presence of hepatic steatosis did not appear to affect the rates of biochemical or histological remission. Even when applying a stricter definition of biochemical remission (ALT and AST < 20 U/L and IgG < 12 g/L), no statistically significant difference was found (Table 5). Logistic regression analysis identified GGT level at the second biopsy as a significant predictor of hepatic steatosis (β = 0.02, OR = 1.02, 95% CI: 1.01–1.03, *p* = 0.031).

**Table 5:** Biochemical and histological characteristics according to hepatic steatosis status at follow-up liver biopsy.

Variables	Total (*n* = 66)	Hepatic steatosis at second liver biopsy (*n* = 21)	No hepatic steatosis at second liver biopsy (*n* = 45)	Statistic	*p*
ALT at the second liver biopsy	14.45 (11.22, 23.38)	15.00 (13.00, 24.60)	13.50 (9.90, 20.10)	*Z* = −1.80	0.072
AST at the second liver biopsy	19.35 (15.85, 30.80)	24.50 (17.80, 31.30)	17.40 (15.10, 22.50)	*Z* = −2.24	0.025
GGT at the second liver biopsy	16.35 (12.22, 31.08)	19.00 (15.00, 69.00)	15.30 (12.00, 23.00)	*Z* = −1.98	0.047
ALP at the second liver biopsy	63.00 (54.00, 82.00)	71.00 (56.00, 87.00)	61.00 (53.00, 82.00)	*Z* = −1.03	0.302
IgG at the second liver biopsy	11.98 ± 3.04	12.15 ± 2.86	11.90 ± 3.15	*t* = −0.31	0.756
TG at the second liver biopsy	1.19 (0.86, 1.43)	1.14 (0.92, 1.39)	1.26 (0.85, 1.53)	*Z* = −0.54	0.591
TC at the second liver biopsy	4.41 ± 0.95	4.65 ± 0.99	4.30 ± 0.92	*t* = −1.42	0.161
Biochemical remission at the second liver biopsy	45 (68.18%)	13 (61.90%)	32 (71.11%)	χ^2^ = 0.56	0.455
Transaminase < 20 and IgG < 12 at the second liver biopsy	25 (37.88%)	7 (33.33%)	18 (40.00%)	χ^2^ = 0.27	0.603
Histological remission				χ^2^ = 0.00	1.000
HAI≥4	15 (22.73%)	5 (23.81%)	10 (22.22%)		
HAI ≤ 3	51 (77.27%)	16 (76.19%)	35 (77.78%)		

## CAP-based assessment of hepatic steatosis progression

Among patients hierarchically assigned to the CAP group, 47 had paired CAP measurements and complete clinical data for the modality-specific analysis. At baseline, eight patients (17.02%) were diagnosed with hepatic steatosis based on their pre-treatment CAP values (≥248 dB/m). After more than 2 years of GCs treatment, 16 patients (34.04%) were diagnosed with hepatic steatosis based on post-treatment CAP values. The McNemar's test showed a statistically significant difference (*p* = 0.039). A significant increase in CAP-defined hepatic steatosis was observed after long-term GCs therapy (Supplementary Table S3).

13 patients developed new or progressive hepatic steatosis, and these patients had significantly higher baseline PLT (159 vs. 113 × 10^9^/L, *p* = 0.048). After 6 months of GCs treatment, their TG levels were elevated (2.01 vs. 1.04 mmol/L, *p* = 0.014; Table 6). Paired *t*-test comparing the pre- and post-treatment CAP values showed a significant increase in CAP after GCs treatment. The average pre-treatment CAP was 191.11 ± 51.39 dB/m, while the post-treatment CAP was 215.45 ± 55.79 dB/m (*t* = −2.46, *p* = 0.018), indicating a significant increase in CAP by an average of 24.34 dB/m (Figure 2E).

**Table 6:** Clinical characteristics according to CAP-defined new-onset or progressive hepatic steatosis among patients with paired CAP measurements (*n* = 47).

Variables	CAP-defined new-onset or progressive steatosis (*n* = 13)	Stable, regressed, or no CAP-defined steatosi (*n* = 34)	*p*
Female (%)	11 (84.62)	26 (76.47)	0.832
Age at disease onset (years)	47.50 (43.00, 57.25)	52.00 (45.00, 57.00)	0.735
BMI	24.37 ± 2.65	22.63 ± 3.20	0.123
Initial dose of glucocorticoids (mg)	24.00 (24.00, 32.00)	24.00 (16.00, 24.00)	0.280
CBR at 6 month (%)	9 (69.23)	21 (56.76)	0.404
ANA (%)	10 (76.92)	27 (79.41)	1.000
SMA (%)	2 (15.38)	3 (8.82)	0.902
Laboratory test at diagnosis			
RBC ( × 10^12^/L)	4.10 (3.92, 4.60)	3.97 (3.51, 4.23)	0.153
Hb (g/L)	121.00 (118.50, 127.75)	121.00 (108.00, 137.00)	0.735
WBC ( × 10^9^/L)	4.46 (3.75, 5.99)	4.50 (3.76, 6.30)	0.907
PLT ( × 10^9^/L)	159.00 (138.50, 188.75)	113.00 (90.00, 164.00)	0.048
ALB (g/L)	38.32 ± 2.96	36.98 ± 6.52	0.351
GLO (g/L)	30.80 (26.12, 34.55)	29.90 (28.20, 34.50)	0.897
ALT (U/L)	215.80 (64.65, 296.27)	147.70 (83.80, 201.60)	0.600
AST (U/L)	187.95 (62.92, 229.55)	128.60 (74.10, 296.50)	0.888
γ-GT (U/L)	158.15 (68.03, 378.25)	126.40 (58.00, 218.00)	0.594
ALP (U/L)	116.50 (89.98, 135.00)	117.20 (96.00, 158.00)	0.524
IgG (g/L)	17.45 ± 6.39	19.69 ± 7.59	0.398
FBG (mmol/L)	5.09 (4.15, 5.52)	4.72 (4.33, 5.50)	0.948
Cholesterol (mmol/L)	3.33 ± 1.01	3.94 ± 1.15	0.135
Triglycerides (mmol/L)	1.51 ± 0.68	1.45 ± 0.62	0.794
High-density lipoprotein (mmol/L)	0.99 (0.46, 1.55)	1.25 (0.86, 1.85)	0.227
Low-density lipoprotein (mmol/L)	1.54 ± 0.85	2.08 ± 0.81	0.068
Laboratory test after 6-month immunosuppressive therapy			
ALT (U/L)	29.60 (19.70, 36.85)	28.60 (19.40, 48.80)	0.659
AST (U/L)	27.00 (22.37, 33.33)	26.00 (21.10, 41.40)	1.000
γ-GT (U/L)	32.00 (22.50, 36.25)	34.00 (20.00, 58.00)	0.559
ALP (U/L)	57.50 (45.75, 62.50)	69.10 (52.00, 94.50)	0.050
FBG (mmol/L)	5.55 ± 1.08	5.37 ± 0.71	0.619
Cholesterol (mmol/L)	6.06 (5.43, 6.38)	5.01 (4.10, 5.79)	0.094
Triglycerides (mmol/L)	2.01 (1.57, 2.76)	1.04 (0.91, 1.84)	0.014
High-density lipoprotein (mmol/L)	1.48 (1.30, 1.93)	1.56 (1.28, 2.20)	0.612
Low-density lipoprotein (mmol/L)	3.65 (2.51, 4.50)	2.74 (1.90, 3.60)	0.172
IgG (g/L)	11.75 (8.32, 15.65)	12.30 (10.55, 14.50)	0.765

When we adjusted the cut-off value to 274 dB/m, we found that 5 patients had hepatic steatosis before treatment, while 14 patients were diagnosed with hepatic steatosis after treatment. The McNemar's test showed a statistically significant difference (*p* = 0.022; Supplementary Table S4). Our analysis shows that the main conclusions drawn using these two cut-off values are consistent, and the impact of different CAP cut-offs on the study's conclusions is minimal.

## Ultrasound findings of hepatic steatosis before and after GCs therapy

A total of 47 patients with complete abdominal ultrasound data before and after treatment were included in the analysis. At baseline, 10 patients (21.28%) were diagnosed with hepatic steatosis. Following GCs treatment, 20 patients (42.55%) were found to have hepatic steatosis, among whom 12 cases represented new-onset steatosis. In contrast, two patients with baseline hepatic steatosis no longer showed steatosis upon follow-up (Figure 2C).

Patients were stratified into two groups based on the presence or absence of new-onset hepatic steatosis. Among the 47 patients, 12 patients (25.53%) developed new-onset hepatic steatosis, while the remaining 35 patients (74.47%) did not. Patients with new-onset steatosis had significantly higher baseline ALT (257.7 vs. 122.9 U/L, *p* = 0.014), while no significant differences were observed in other clinical parameters (Supplementary Table S5).

To evaluate changes in steatosis status before and after treatment, McNemar's test was performed (Supplementary Table S6). Among the 47 patients, two transitioned from a positive to a negative status for steatosis, while 12 transitioned from a negative to a positive status. The analysis indicated that patients were more likely to develop hepatic steatosis after GCs treatment (*p* = 0.0129).

## Six-month triglyceride levels as an early metabolic marker

To identify early metabolic markers of new-onset or progressive hepatic steatosis, univariate and multivariate logistic regression analyses were conducted in the entire cohort of patients with AIH, as well as in subgroups with paired liver histology, CAP, or ultrasound data.

In the entire AIH cohort (Table 7), multivariate analysis revealed that female sex (OR = 4.93, 95% CI: 1.20–20.24, *p* = 0.027), higher RBC count at diagnosis (OR = 3.22, 95% CI: 1.26–8.23, *p* = 0.015), and elevated TG levels at 6 months (OR = 2.41, 95% CI: 1.49–3.88, *p* < 0.001) were independently associated markers of hepatic steatosis progression. Although baseline GCs dose, FBG, TC, and ALB were statistically significant in univariate analysis, they were not retained in the multivariate model. In exploratory interaction analysis (Supplementary Table S13), the interaction between TG after 6 months of treatment and BMI at diagnosis showed a weak positive trend.

**Table 7:** Factors associated with new-onset or progressive hepatic steatosis after treatment in the entire AIH cohort.

Variables	Univariate analysis		Multivariate analysis	
	OR (95%CI)	*p* value	OR (95%CI)	*p* value
Female	2.17 (0.78 ~ 6.08)	0.14	4.93 (1.20 ~ 20.24)	0.027
Initial dose of glucocorticoids	1.06 (1.01 ~ 1.13)	0.033		
RBC at diagnosis	2.91 (1.46 ~ 5.79)	0.002	3.22 (1.26 ~ 8.23)	0.015
WBC at diagnosis	1.08 (0.98 ~ 1.18)	0.103	1.02 (0.95 ~ 1.10)	0.54
ALB at diagnosis	1.09 (1.01 ~ 1.18)	0.021		
ALT at diagnosis	1.00 (1.00 ~ 1.00)	0.09	1.00 (1.00 ~ 1.00)	0.082
ALP at diagnosis	1.00 (0.99 ~ 1.00)	0.177	0.99 (0.98 ~ 1.00)	0.057
BMI at diagnosis	1.09 (0.98 ~ 1.22)	0.121		
TG at diagnosis	1.14 (0.77 ~ 1.69)	0.502		
ALP after 6 months of treatment	0.98 (0.97 ~ 0.99)	0.047		
FBG after 6 months of treatment	1.53 (1.02 ~ 2.30)	0.042		
TC after 6 months of treatment	1.57 (1.19 ~ 2.06)	0.001		
TG after 6 months of treatment	2.48 (1.62 ~ 3.79)	< 0.001	2.41 (1.49 ~ 3.88)	< 0.001

In a sensitivity analysis forcing BMI at diagnosis and baseline TG into the multivariable model, TG after 6 months of treatment remained independently associated with new-onset or progressive hepatic steatosis, whereas baseline TG and BMI were not independently associated with the endpoint (Supplementary Table S12).

In the subgroup of patients who underwent paired liver biopsies (Table 8), increased RBC count at diagnosis (OR = 8.76, 95% CI: 1.12–68.74, *p* = 0.039) and elevated TG levels after treatment (OR = 3.35, 95% CI: 1.40–8.00, *p* = 0.006) were independently associated with steatosis progression.

**Table 8:** Predictors of new-onset or progressive hepatic steatosis after treatment in AIH patients with twice liver histopathological examination.

Variables	Univariate analysis		Multivariate analysis	
	OR (95%CI)	*p* value	OR (95%CI)	*p* value
FBG at diagnosis	1.36 (0.86 ~ 2.17)	0.193	1.58 (0.90 ~ 2.77)	0.112
RBC at diagnosis	7.07 (1.95 ~ 25.64)	0.003	8.76 (1.12 ~ 68.74)	0.039
Hb at diagnosis	1.05 (1.01 ~ 1.10)	0.009		
WBC at diagnosis	1.15 (1.00 ~ 1.32)	0.05	1.05 (0.81 ~ 1.38)	0.699
GLO at diagnosis	1.10 (1.01 ~ 1.20)	0.041	1.18 (0.98 ~ 1.42)	0.082
ALT after 6 months of treatment	0.97 (0.93 ~ 1.01)	0.127	0.93 (0.85 ~ 1.01)	0.074
FBG after 6 months of treatment	1.55 (0.83 ~ 2.89)	0.168	2.90 (0.78 ~ 10.72)	0.111
TG after 6 months of treatment	2.93 (1.45 ~ 5.91)	0.003	3.35 (1.40 ~ 8.00)	0.006

In patients with pre-treatment and post-treatment CAP measurements (Table 9), higher body mass index (BMI: OR = 1.56, 95% CI: 1.04–2.35, *p* = 0.032) and elevated TG levels after 6-month treatment (OR = 8.41, 95% CI: 1.41–50.38, *p* = 0.020) emerged as independently associated markers. Exploratory interaction analysis did not show a significant interaction between TG after 6 months of treatment and baseline CAP. After adjustment for BMI, the interaction term was not statistically significant (OR = 1.14, 95% CI: 0.93–1.40, *p* = 0.211; likelihood-ratio test *p* for interaction = 0.194).

**Table 9:** Predictors of new-onset or progressive hepatic steatosis after treatment in AIH patients who underwent CAP measurements before and after GCs treatment.

Variables	Univariate analysis		Multivariate analysis	
	OR (95%CI)	*p* value	OR (95%CI)	*p* value
BMI at diagnosis	1.23 (0.94 ~ 1.60)	0.128	1.56 (1.04 ~ 2.35)	0.032
TC at diagnosis	0.59 (0.30 ~ 1.19)	0.14	0.31 (0.07 ~ 1.35)	0.119
Low-density lipoprotein at diagnosis	0.42 (0.16 ~ 1.10)	0.077	0.24 (0.05 ~ 1.23)	0.088
PLT at diagnosis	1.01 (1.00 ~ 1.02)	0.171	1.01 (1.00 ~ 1.03)	0.094
ALP after 6 months of treatment	0.96 (0.92 ~ 1.00)	0.067	0.95 (0.88 ~ 1.02)	0.14
TG after 6 months of treatment	2.36 (1.07 ~ 5.21)	0.034	8.41 (1.41 ~ 50.38)	0.02

Among patients who underwent abdominal ultrasound both before and after treatment (Supplementary Table S7), elevated ALT at diagnosis (OR = 1.01, 95% CI: 1.01–1.01, *p* = 0.023) and lower ALP (OR = 0.96, 95% CI: 0.92–0.99, *p* = 0.021) were significantly associated with new-onset hepatic steatosis.

Notably, elevated TG levels at 6 months after treatment consistently emerged as an independently associated marker of new-onset or progressive hepatic steatosis across multiple subgroups. Specifically, TG was a significant independent risk factor in the entire AIH cohort (OR = 2.41, 95% CI: 1.49–3.88, *p* < 0.001), in patients who underwent two liver biopsies (OR = 3.35, 95% CI: 1.40–8.00, *p* = 0.006), and in those who received CAP assessments both before and after treatment (OR = 8.41, 95% CI: 1.41–50.38, *p* = 0.020).

ROC analysis further demonstrated that TG levels at 6 months had moderate predictive value for the development or progression of hepatic steatosis in the entire cohort (AUC = 0.723), the biopsy subgroup (AUC = 0.725), and the CAP subgroup (AUC = 0.758). The optimal TG cutoff values were 1.615 mmol/L (sensitivity: 62.8%, specificity: 74.4%) in the entire cohort, 2.445 mmol/L (sensitivity: 44.4%, specificity: 95.8%) in the biopsy cohort, and 1.445 mmol/L (sensitivity: 90.0%, specificity: 64.9%) in the CAP cohort (Figure 2F). These findings suggest that post-treatment hypertriglyceridemia may be an important marker associated with the development and progression of hepatic steatosis in AIH patients across different assessment modalities.

## Component analysis of new-onset and progressive hepatic steatosis

To clarify the composition of the composite endpoint, we further separated new-onset steatosis from progression of pre-existing steatosis (Supplementary Table S9). Among the 43 patients who met the composite endpoint, 35 had new-onset hepatic steatosis and eight had progression of pre-existing steatosis. In the liver biopsy subgroup, 18 patients met the composite endpoint, including 13 new-onset events and five progression events. In the CAP subgroup, 13 patients met the composite endpoint, including 10 new-onset events and three progression events defined by an increase in CAP of ≥20 dB/m among patients with baseline CAP-defined steatosis. In the ultrasound subgroup, 12 patients developed new-onset steatosis. Because routine ultrasound was recorded as a binary assessment, ultrasound-defined progression of pre-existing steatosis was not quantified.

For the new-onset component, the analysis was restricted to patients without hepatic steatosis at baseline. In multivariable logistic regression (Supplementary Table S10), 6-month triglyceride levels remained independently associated with new-onset hepatic steatosis (OR = 2.78, 95% CI: 1.61–4.78, *p* < 0.001). Baseline RBC count was also associated with new-onset steatosis (OR = 2.43, 95% CI: 1.02–5.81, *p* = 0.046), whereas 6-month ALP showed only a borderline inverse association (OR = 0.98, 95% CI: 0.97–1.00, *p* = 0.071).

For progression of pre-existing steatosis, only eight events were observed. Therefore, conventional multivariable logistic regression was not performed for this component. In exploratory analyses, patients with progression tended to have higher BMI and higher 6-month LDL-C levels in descriptive comparisons, but univariable logistic regression did not identify robust progression-specific predictors (Supplementary Table S11).

## Consistency and sensitivity comparison of biopsy, CAP, and ultrasound in diagnosing hepatic steatosis

Among these 160 patients, 99 had paired data from more than one assessment method, and 26 had paired data from all three assessment methods. We conducted a consistency analysis on 26 patients who had all three test results (Supplementary Figure S1). Cohen's Kappa was calculated to assess the consistency between different diagnostic methods. Pre-treatment, the Kappa value between liver biopsy and CAP was 0.82. The agreement between CAP and ultrasound was excellent (Kappa = 0.91).

The sensitivity for diagnosing hepatic steatosis with CAP was 87.5%, and the specificity was 94.4%. The sensitivity for ultrasound diagnosis was 75%, with a specificity of 94.4%. Post-treatment, the three methods also showed high overall consistency (Supplementary Table S8), with CAP showing a sensitivity of 92.31% and specificity of 100%. The sensitivity for ultrasound diagnosis was 84.62%, with a specificity of 92.31%.

## Discussion

Currently, GCs remain the first-line treatment for AIH (1). GCs exert their therapeutic effect primarily by suppressing aberrant immune activation, thereby reducing hepatic inflammation and promoting the restoration of liver function. Although GCs effectively control liver inflammation, long-term use, even at low doses, may lead to a range of metabolic complications, including dyslipidemia, hyperglycemia, weight gain, osteoporosis, and hepatic steatosis (3, 4, 17–19).

Research on the adverse effects of GCs has predominantly focused on rheumatic diseases. However, AIH also necessitates long-term immunosuppressive therapy, yet there are few studies specifically addressing GC-related adverse outcomes in AIH. A study conducted in the Netherlands indicated that even long-term, low-dose GCs therapy (10 mg/day or less) may increase the risk of cataracts, osteoporotic fractures, and diabetes in patients with AIH (20). A study by Jalal et al. demonstrated that AIH patients have a significantly increased risk of developing diabetes, hypertension, dyslipidemia, and elevated BMI. However, that study did not specifically focus on the impact of GCs on metabolic disorders (21).

In our entire cohort, 26.88% of AIH patients developed new-onset or progressive hepatic steatosis after more than 24 months of GCs therapy. This finding indicates that more than one-quarter of patients may be at increased risk of developing hepatic steatosis during long-term GCs therapy.

Further analyses based on different clinical assessment modalities showed consistent trends. Among patients who underwent two liver biopsies, 27.17% exhibited new-onset or progressive hepatic steatosis. In those with longitudinal CAP measurements, the proportion was 21.27%, while among patients who received pre- and post-treatment ultrasound evaluations, 25.53% demonstrated steatosis development or progression.

Although slight variations were observed among assessment modalities, the overall trend was consistent across methods, supporting an association between long-term GCs therapy and hepatic steatosis development or progression in patients with AIH.

Liver biopsy remains the gold standard for the diagnosis and evaluation of various liver diseases. In the histological subgroup, the prevalence of hepatic steatosis increased from 21.21% at baseline to 31.82% after GCs therapy. Notably, 27.17% of patients developed new-onset or progressive steatosis following treatment. Although there was a clear upward trend in hepatic steatosis during GCs therapy, the association between repeated liver biopsy findings and the incidence of steatosis progression was not statistically significant (*p* = 0.167). This non-significant result should be interpreted cautiously and should not be considered definitive evidence of no histological change. The biopsy subgroup included only 66 patients, and only 19 discordant pairs contributed directly to the McNemar test, which substantially limited statistical power. Therefore, the biopsy findings suggest a possible trend toward increased hepatic steatosis during long-term glucocorticoid therapy, but the sample size was insufficient to exclude either no true difference or a clinically meaningful increase.

This non-significant result may be attributed to the relatively small sample size. In addition, although liver histopathology remains the diagnostic gold standard for hepatic steatosis, it is subject to sampling variability, which may result in underestimation or even missed detection of mild steatosis depending on the biopsy site. Furthermore, the influence of potential confounding factors that were not captured in this study cannot be entirely ruled out. For example, AIH patients with steatosis identified at the time of the first biopsy may have paid greater attention to diet, exercise, and body weight management in daily life, thereby exhibiting heightened awareness of GCs-related metabolic complications.

In this study, the mean CAP value increased by 24.34 dB/m after treatment (*p* = 0.018), indicating an increase in hepatic fat content. The prevalence of hepatic steatosis rose from 17.02% before GCs therapy to 34.04% after treatment. Furthermore, the McNemar's test (*p* = 0.039) supported this observation. Furthermore, when we adjusted the cut-off value to 274 dB/m and conducted further sensitivity analysis, the results remained consistent.

Similarly, among 47 patients who underwent abdominal ultrasound examinations both before and after treatment, the detection rate of hepatic steatosis increased from 21.28 to 42.55%, with 12 patients of new-onset steatosis and only two patients showing regression. These findings further indicate an increased risk of developing hepatic steatosis following GCs treatment (*p* = 0.0129).

Hepatic fat accumulation may occur during long-term GCs therapy even when hepatic inflammation is well controlled. This observation may be biologically plausible, given the known lipogenic potential of GCs, particularly with extended exposure. Extensive basic research has demonstrated that GCs can promote *de novo* lipogenesis and TG accumulation by binding to glucocorticoid response elements in key lipogenic genes via the glucocorticoid receptor, leading to the upregulation of sterol regulatory element binding protein-1c, fatty acid synthase, acetyl-CoA carboxylase 1, and stearoyl-CoA desaturase-1 (22–24). Concurrently, GCs have been shown to inhibit fatty acid oxidation, thereby reducing hepatic lipid utilization and promoting microvesicular steatosis (25). However, no studies have clearly distinguished classical NAFLD from steroid-induced hepatic steatosis.

Further analysis of predictive factors revealed that patients who developed new-onset or progressive hepatic steatosis exhibited more pronounced lipid metabolism disturbances as early as 6 months into GCs therapy. Notably, elevated TG levels at 6 months consistently emerged as an independently associated marker of hepatic steatosis progression across multiple subgroup cohorts.

Although baseline TG and BMI are clinically relevant metabolic variables, neither was independently associated with the composite endpoint in the sensitivity analysis. In contrast, 6-month TG remained significantly associated with hepatic steatosis after adjustment for these baseline factors, suggesting that it may reflect an early dynamic metabolic response during glucocorticoid therapy rather than simply baseline metabolic susceptibility.

In addition, the component-specific analysis provided further insight into the composite endpoint. Most events were attributable to new-onset steatosis rather than progression of pre-existing steatosis. In particular, 6-month triglyceride levels remained strongly associated with new-onset hepatic steatosis, suggesting that early lipid metabolic disturbance during glucocorticoid therapy may be more closely related to the development of *de novo* hepatic fat accumulation. By contrast, progression of pre-existing steatosis occurred in only eight patients, limiting the statistical power to identify reliable progression-specific predictors. Although patients with progression showed higher BMI and lipid parameters in descriptive analyses, the corresponding effect estimates were imprecise. Therefore, the progression-specific findings should be interpreted as exploratory. These results support the use of the composite endpoint for the primary analysis while clarifying that the main metabolic signal, especially 6-month triglyceride elevation, was largely driven by the new-onset steatosis component.

These findings suggest that early metabolic dysregulation may play a pivotal role in the new-onset and progression of GCs-associated hepatic steatosis, with elevated TG levels at 6 months potentially reflecting a sustained lipid metabolic burden. Previous studies have demonstrated a strong association between TG levels and both the onset and progression of NAFLD (26, 27). Moreover, plasma TG levels are positively and dose-dependently associated with both the risk and severity of NAFLD (28, 29). Specific alleles in TG-regulating genes—such as APOA5, APOC3, and TM6SF2—have also been associated with an increased risk and severity of NAFLD, as well as the regulation of circulating and intrahepatic TG levels (30, 31).

Traditionally, BMI is considered to be positively correlated with hepatic steatosis. In our study, an increased BMI emerged as an independently associated marker of hepatic steatosis in the CAP sub-group. In the secondary liver biopsy cohort, we found that patients with new-onset hepatic steatosis might exhibit an increase in BMI as early as 6 months. Exploratory interaction analyses suggested only a weak positive trend for the interaction between 6-month TG and BMI, whereas no significant interaction was observed between 6-month TG and baseline CAP in the paired CAP subgroup. Although biological interaction among TG, BMI, and baseline hepatic fat burden is plausible, these findings do not provide definitive evidence of effect modification and should be interpreted cautiously because of the limited number of outcome events.

Interestingly, in the liver histology cohort, patients with new-onset or progressive hepatic steatosis had significantly higher baseline levels of RBC, WBC, Hb, ALB, and GLO compared to other patients. We observed similar trends in the entire cohort (RBC, PLT, ALB), as well as in the CAP group, where the PLT level was also elevated. These parameters are not traditionally associated with liver function or lipid metabolism and are often overlooked in prior studies. In patients with chronic liver disease, elevated levels of RBC, WBC, Hb, and ALB typically reflect better hepatic reserve function and nutritional status.

Female sex and higher baseline RBC count were also associated with new-onset or progressive hepatic steatosis in the multivariable model. These associations should be interpreted cautiously and should not be considered direct causal relationships. The association with female sex may partly reflect sex-related metabolic susceptibility during glucocorticoid therapy. In particular, peri-menopausal or post-menopausal women may be more vulnerable to changes in fat distribution, insulin sensitivity, and lipid metabolism, which could facilitate hepatic fat accumulation during long-term glucocorticoid exposure.

AIH patients with better baseline nutritional and metabolic profiles may be more susceptible to hepatic steatosis during long-term GCs therapy. In contrast, patients with portal hypertension may exhibit lower RBC, WBC, and PLT levels due to hypersplenism, which is often accompanied by impaired hepatic synthetic function and pre-existing disturbances in lipid metabolism (32). Furthermore, elevated RBC and Hb levels are commonly associated with individuals who have higher BMI, insulin resistance, or metabolic syndrome, indicating possible shared metabolic characteristics. Previous studies have demonstrated that WBC count and its subtypes can reliably predict the prevalence and future incidence of NAFLD, independent of classical metabolic risk factors (33–35). RBC and Hb have been shown to independently predict the occurrence and prevalence of NAFLD (36–39). However, it should be specifically noted that the baseline RBC count should be regarded as a clinical surrogate marker for hepatic steatosis, rather than a mechanistic driver.

In certain liver diseases, hepatocellular fat accumulation may occur as part of the tissue repair process following inflammatory injury. Some studies have proposed a biological hypothesis termed “inflammation–repair–steatosis” to explain this phenomenon (40). However, in our study, baseline levels of ALT, AST, and ALP were not significantly different between patients with steatosis progression and other patients. The finding suggests that hepatic fat accumulation observed in our cohort is unlikely to be a direct consequence of AIH-related inflammatory activity or subsequent tissue repair. Instead, it is more likely attributable to favorable nutritional status and early disturbances in lipid metabolism. Notably, dysregulation of lipid metabolism in AIH patients appears to emerge during the early phase of immunological remission following the initiation of GCs therapy.

Elevated GGT and AST levels in patients with hepatic steatosis at the time of the second liver biopsy may reflect subclinical liver injury or oxidative stress induced by fat accumulation. Among these markers, GGT demonstrated not only a statistically significant elevation in the steatosis group but also emerged as an independent predictor in logistic regression analysis (β = 0.02, OR = 1.02, 95% CI: 1.01–1.03, *p* = 0.031). Interestingly, although GGT and AST levels were elevated, other metabolic parameters such as TC, TG, and IgG did not differ significantly between patients with or without steatosis. Moreover, the presence of steatosis did not appear to compromise biochemical or histological remission, even when more stringent remission criteria (ALT and AST < 20 U/L and IgG < 12 g/L) were applied. This dissociation between fat accumulation and inflammatory control highlights the possibility that metabolic injury may occur independently of immune-mediated disease activity.

These findings highlight the importance of identifying an optimal “balanced dose” of GCs—one that effectively suppresses hepatic immune-mediated inflammation while minimizing the risk of hepatic fat accumulation. Achieving this balance may require careful tapering strategies or the use of combination therapy with other immunosuppressive agents.

Furthermore, patients with AIH should undergo dynamic monitoring for hepatic steatosis. In particular, regular non-invasive assessments—such as FibroScan—are recommended for those who develop metabolic risk factors during treatment. These measures may facilitate the early detection of metabolic liver injury, allowing for timely adjustment of immunosuppressive regimens and the initiation of lifestyle interventions (1).

This study is a single-center, retrospective cohort study with a limited sample size, and there are some limitations. First, the sensitivity and specificity of different methods used to assess hepatic steatosis may vary. Although we employed a grading strategy—using histological examination as the gold standard to minimize bias—measurement errors could not be completely eliminated. Variations attributable to differences in operators or interpreters may occur during CAP and ultrasound assessments. Second, the GCs dose in AIH patients is highly individualized and requires dynamic adjustments during treatment. Therefore, this study only recorded the induction dose and was unable to quantify the cumulative exposure to GCs, which limits the assessment of the dose-response relationship. Our team is currently investigating the impact of cumulative GCs exposure on clinical adverse reactions in AIH patients. Additionally, although we informed all enrolled patients to maintain their usual lifestyle with respect to diet and activity, and excluded patients with a history of heavy alcohol consumption from the analysis, the lack of data on other lifestyle factors may still influence the accuracy of the study's results. In addition, this study did not systematically record the patients' menopausal status, hypersplenism, portal hypertension, or dynamic changes in BMI. Furthermore, selection bias arising from the lack of paired assessments of steatosis or incomplete biochemical data cannot be entirely ruled out.

Finally, since the standard for CAP diagnosis of hepatic steatosis has not been unified, we used 248 dB/m as the diagnostic threshold for CAP in this study. Karla T et al. (11) reported that in a study involving 2735 patients with chronic liver disease of different etiologies, the optimal CAP cut-off for ≥S1 (steatosis present vs. absent) was 248 dB/m. A meta-analysis by Roxana et al. (12) also showed that the best cut-off for ≥S1 was 248 dB/m, with a sensitivity of 0.68 and specificity of 0.82. The thresholds of 268 dB/m and 280 dB/m can be used to diagnose ≥S2 and S3, respectively. On one hand, this is because the AIH patient population represents an atypical NAFLD background, and their baseline risk of developing steatosis may be lower than that of conventional NAFLD cohorts. Duarte et al. (41) used “≥248 dB/m” to define steatosis (S1) in HIV-infected individuals. On the other hand, this threshold was chosen to increase the sensitivity for detecting early mild fatty liver changes. Furthermore, we explored the sensitivity results using ≥274 dB/m as the CAP threshold in a supplementary analysis and ultimately reached the same trend.

There is currently limited research on the longitudinal changes of CAP values. In this study, we used an increase in CAP ≥20 dB/m as the threshold for analyzing progression based on clinical judgment and existing literature. In a longitudinal study conducted by Cervera et al. (42), the new onset of hepatic steatosis was defined as a >10% increase in CAP with a final CAP ≥275 dB/m.

Prolonged exposure to GCs may not only contribute to hepatic fat accumulation but also to complications such as osteoporosis, gastrointestinal ulceration, and dysregulation of glucose and lipid metabolism. Future studies are warranted to explore these adverse effects further. Persistent GCs-related hepatic fat accumulation may adversely impact both metabolic health and liver function in AIH patients. Although GCs are effective in suppressing autoimmune activity and resolving hepatic inflammation, prolonged lipid accumulation could paradoxically exacerbate liver injury and contribute to the development of metabolic dysfunction-associated steatotic liver disease.

Multiple studies have reported that coexisting NAFLD or nonalcoholic steatohepatitis in patients with AIH is associated with a suboptimal treatment response. Hepatic steatosis is recognized as a negative prognostic marker, strongly associated with increased risk of fibrosis and cirrhosis progression (7, 8, 43). However, no studies have focused explicitly on whether steroid-related hepatic steatosis during long-term low-dose GCs maintenance therapy impacts long-term outcomes in AIH patients. Future longitudinal follow-up of this cohort is warranted to investigate patient outcomes and disease progression under the combined burden of AIH and GCs-associated hepatic steatosis.

In conclusion, long-term GCs therapy may be associated with the development and progression of hepatic steatosis in patients with AIH. Multimodal assessment strategies are of considerable value in the early detection of individuals at increased risk. Notably, TG level at 6 months post-treatment emerged as a significant predictor of steatosis onset and progression. In this cohort, hepatic steatosis progression was not clearly associated with biochemical indicators of immune response. In the management of AIH, clinicians should strive to balance the immunosuppressive efficacy of GCs with their potential metabolic adverse effects, particularly those related to lipid metabolism.

## Data Availability

The datasets analyzed during the current study are not publicly available due to patient privacy, ethical restrictions, and institutional data governance policies. Requests to access the datasets should be directed to the corresponding author and will be considered in accordance with institutional regulations.
