# Supplementary material for: Development and progression of hepatic steatosis during long-term glucocorticoid therapy in autoimmune hepatitis: multimodal evidence from biopsy, CAP, and ultrasound
Source: Front Med (Lausanne). 2026 Aug 5;13:1891955. doi: 10.3389/fmed.2026.1891955 (PMC13485760; doi:10.3389/fmed.2026.1891955)
Supplement: Supplementary file 1 [file Table_1.docx]

**Fig. 1. Flowchart of patient selection and subgroup classification.**

A total of 946 patients diagnosed with autoimmune hepatitis (AIH) with liver histology available at the Second Hospital of Nanjing between 2017 and 2023 were screened. After excluding patients with coexisting PBC, PSC, IgG4-SC, or other chronic liver diseases (n = 586), those who received <24 months of GCs therapy (n = 97), were treated with budesonide (n = 6), had long-term prior use of GCs for other diseases (n = 4), were younger than 18 years (n = 2), had heavy alcohol consumption (n = 8), or had incomplete clinical data (n = 11), 232 patients were assessed. An additional 72 patients were excluded because paired assessment of hepatic steatosis before and after GCs therapy by liver histology, CAP, or ultrasound was unavailable. Ultimately, 160 patients were included in the final analysis. Among these patients, paired liver histology, paired CAP measurements, and paired abdominal ultrasound data were available in 66, 77, and 142 patients, respectively. For the hierarchical overall analysis, patients were assigned to the biopsy group (n = 66), CAP group (n = 47), or ultrasound group (n = 47).

**Fig. 2. Multimodal Evaluation of Steatosis Progression and Its Predictors in AIH Patients During Glucocorticoid Therapy**

(A) Distribution changes of hepatic steatosis status in 66 AIH patients based on initial and follow-up liver biopsies. Bars are divided into Pure AIH and AIH with steatosis, illustrating the progression of steatosis during follow-up.
(B) Distribution of fibrosis trends according to hepatic steatosis dynamics in patients with serial liver biopsies.
(C) Sankey diagram showing changes in ultrasound-detected hepatic steatosis before and after glucocorticoid therapy.
(D) Paired changes in histological activity index (HAI) between the initial and follow-up liver biopsies, showing a significant decrease during follow-up.

(E) Paired changes in controlled attenuation parameter (CAP) before and after glucocorticoid therapy, showing a significant increase after treatment.
(F) Receiver operating characteristic (ROC) curves showing the predictive performance of 6-month triglyceride levels for new-onset or progressive hepatic steatosis in the overall cohort, biopsy cohort, and CAP cohort.

**Fig. 3. Representative paired liver biopsy sections showing progression of hepatic steatosis during long-term GCs therapy in patients with autoimmune hepatitis.**

(A) Baseline liver biopsy specimen from a patient with AIH before GCs therapy.
(B) Follow-up liver biopsy specimen from the same patient after long-term GCs therapy, showing increased hepatocellular steatosis.

Sections were stained with hematoxylin and eosin (H&E).

**Fig. 4. Dynamic changes in metabolic parameters in AIH patients with paired liver biopsies during glucocorticoid therapy**

HDL-C, LDL-C, BMI, UA, FBG, TC, and TG were evaluated at baseline, at 6 months after glucocorticoid therapy, and at the time of the second liver biopsy in AIH patients with paired liver biopsy data. Pairwise comparisons between time points are shown above each panel. ns indicates no significant difference; *P < 0.05, **P < 0.01, and ****P < 0.0001.

**Fig. S1. Overlap among patients with paired liver biopsy, CAP, and ultrasound data.**

Venn diagram showing the overlap among patients with paired liver biopsy, CAP, and ultrasound data in the final cohort (N = 160). Overall, 99 patients had paired data from more than one assessment modality, and 26 patients had paired data from all three modalities.
