# Supplementary material for: Development and progression of hepatic steatosis during long-term glucocorticoid therapy in autoimmune hepatitis: multimodal evidence from biopsy, CAP, and ultrasound
Source: Front Med (Lausanne). 2026 Aug 5;13:1891955. doi: 10.3389/fmed.2026.1891955 (PMC13485760; doi:10.3389/fmed.2026.1891955)
Supplement: Supplementary file 2 [file Table_2.docx]

**Supplementary Table 1. Clinical characteristics of the overall cohort.**

| **Variables** | **Total (n = 160)** | **Liver histopathological examination**  **(n = 66)** | **CAP**  **(n = 47)** | **Liver ultrasound examination**  **(n = 47)** | ***P*** |
| --- | --- | --- | --- | --- | --- |
|  |  |  |  |  |  |
| **Female, n(%)** | 129 (80.62) | 53 (80.30) | 37 (78.72) | 39 (82.98) | 0.869 |
| **Age** | 53.00 (45.00, 60.00) | 53.00 (47.25,60.00) | 52.00 (43.00,57.00) | 56.00 (45.00,63.50) | 0.256 |
| **BMI** | 22.95 (21.04, 24.79) | 23.65 (21.62,25.32) | 23.39 (20.62,25.17) | 22.10 (20.97,23.68) | 0.083 |
| **Initial dose of glucocorticoids(mg)** | 24.00 (20.00, 32.00) | 24.00 (24.00,32.00) | 24.00 (18.00,31.00) | 24.00 (16.00,30.00) | 0.328 |
| **CBR at 6 month,n（%）** | 94 (58.75) | 40 (60.61) | 28 (59.57) | 26 (55.32) | 0.846 |
| **ANA, n(%)** | 113 (70.62) | 45 (68.18) | 37 (78.72) | 31 (65.96) | 0.338 |
| **SMA, n(%)** | 15 (9.38) | 7 (10.61) | 5 (10.64) | 3 (6.38) | 0.787 |
| **Laboratory test at diagnosis** | | | | | |
| **RBC（×10^12^/L)** | 4.01 (3.68, 4.33) | 4.07 (3.80,4.37) | 4.00 (3.67,4.33) | 3.92 (3.62,4.19) | 0.119 |
| **WBC（×10^9^/L)** | 4.66 (3.70, 8.02) | 4.85 (3.66,8.14) | 4.47 (3.71,6.23) | 5.18 (3.77,9.72) | 0.258 |
| **Hb(g/L)** | 124.95 ± 17.72 | 127.28 ± 17.22 | 124.23 ± 19.28 | 122.38 ± 16.70 | 0.334 |
| **PLT（×10^9^/L)** | 135.50 (96.00, 178.00) | 143.50 (102.50,180.50) | 126.00 (95.00,169.00) | 133.00 (97.00,178.50) | 0.510 |
| **GLO(g/L)** | 29.20 (25.93, 34.78) | 28.30 (24.70,31.85) | 30.00 (27.95,34.75) | 29.50 (26.30,35.80) | 0.095 |
| **ALB(g/L)** | 37.86 ± 4.98 | 39.07 ± 4.42 | 37.26 ± 5.94 | 36.74 ± 4.38 | **0.030** |
|  |  |  |  |  |  |
| **ALT(U/L)** | 110.30 (48.45, 277.75) | 62.50 (17.07,287.33) | 167.30 (74.50,267.30) | 153.30 (88.45,283.10) | **0.005** |
| **AST(U/L)** | 117.30 (52.88, 276.65) | 71.00 (24.12,164.38) | 131.30 (66.05,288.45) | 189.30 (64.65,318.15) | **<0.001** |
| **γ-GT(U/L)** | 96.30 (47.92, 175.95) | 75.50 (24.35,130.60) | 131.00 (62.85,220.00) | 117.50 (59.50,191.25) | **<0.001** |
| **ALP(U/L)** | 114.50 (82.88, 154.15) | 97.25 (73.25,149.50) | 117.20 (93.70,148.85) | 119.00 (91.15,158.35) | 0.124 |
| **IgG(g/L)** | 16.15 (12.17, 19.83) | 14.95 (11.48,17.61) | 18.20 (13.25,24.35) | 16.40 (13.03,20.20) | **0.008** |
| **FBG(mmol/L)** | 4.83 (4.38, 5.45) | 4.76 (4.45,5.33) | 4.78 (4.29,5.52) | 4.87 (4.33,5.45) | 0.984 |
| **Cholesterol(mmol/L)** | 3.89 ± 1.19 | 4.05 ± 1.20 | 3.81 ± 1.14 | 3.75 ± 1.23 | 0.364 |
| **Triglycerides(mmol/L)** | 1.33 (0.93, 1.90) | 1.26 (1.01,2.10) | 1.37 (0.95,1.88) | 1.35 (0.88,1.81) | 0.851 |
| **High-density lipoprotein(mmol/L)** | 1.10 (0.78, 1.69) | 1.10 (0.79,1.53) | 1.25 (0.81,1.79) | 1.02 (0.71,1.78) | 0.575 |
| **Low-density lipoprotein(mmol/L)** | 2.05 ± 0.82 | 2.19 ± 0.80 | 1.97 ± 0.84 | 1.92 ± 0.82 | 0.182 |
| **Laboratory test after six-month immunosuppressive therapy** | | | | | |
| **ALT(U/L)** | 24.25 (18.03, 38.12) | 23.60 (16.92,35.05) | 28.60 (19.05,45.97) | 24.30 (17.65,37.40) | 0.147 |
| **AST(U/L)** | 26.60 (20.75, 37.35) | 26.10 (19.88,35.20) | 26.00 (21.15,40.39) | 27.50 (20.75,40.40) | 0.788 |
| **γ-GT(U/L)** | 27.00 (16.90, 47.62) | 21.65 (15.00,45.23) | 32.00 (20.00,50.67) | 24.00 (17.30,43.77) | 0.319 |
| **ALP(U/L)** | 66.05 (53.15, 83.33) | 63.30 (51.25,83.48) | 63.00 (50.60,84.50) | 68.00 (57.30,81.27) | 0.525 |
| **FBG(mmol/L)** | 5.21 (4.75, 5.82) | 5.04 (4.62,5.64) | 5.34 (4.81,5.92) | 5.24 (4.82,5.94) | 0.361 |
| **Cholesterol(mmol/L)** | 5.01 (4.12, 5.91) | 4.98 (4.13,5.82) | 5.18 (4.17,6.30) | 4.85 (4.00,5.62) | 0.385 |
| **Triglycerides(mmol/L)** | 1.35 (0.91, 1.90) | 1.42 (0.86,1.94) | 1.36 (0.95,2.12) | 1.14 (0.90,1.71) | 0.467 |
| **High-density lipoprotein(mmol/L)** | 1.60 (1.31, 2.03) | 1.70 (1.38,2.07) | 1.56 (1.29,2.20) | 1.52 (1.30,1.85) | 0.351 |
| **Low-density lipoprotein(mmol/L)** | 2.75 (2.17, 3.63) | 2.74 (2.25,3.32) | 2.96 (2.01,3.89) | 2.71 (2.17,3.55) | 0.872 |
| **IgG(g/L)** | 12.05 (10.10, 14.59) | 11.90 (9.72,14.35) | 12.30 (9.80,14.65) | 12.00 (10.41,14.57) | 0.754 |

**Supplementary Table 2.The changes of hepatic steatosis in patients with hepatic steatosis both before and after treatment(n = 8).**

| **Number** | **Percentage of hepatic steatosis** | | | **Hepatic Steatosis Distribution Patterns** | | | | | **Steatosis Type** | |
| --- | --- | --- | --- | --- | --- | --- | --- | --- | --- | --- |
|  | **Pre-treatment** | **Post-treatment** | **Hepatic steatosis status** | **Pre-treatment** | | | **Post-treatment** | | **Pre-treatment** | **Post-treatment** |
| 1 | 25 | 20 | stable | focal | zone 3 and some periportal areas | diffuse | | zone 3 and some periportal areas | macrovesicular | macrovesicular |
| 2 | 5 | 35 | marked progression | diffuse | zone 3 | | focal | zone 3 | macrovesicular | mixed，mainly macrovesicular |
| 3 | 10 | 35 | moderate progression | diffuse | zone 3 | | mixed | zone 3 | macrovesicular | macrovesicular |
| 4 | 15 | 5 | stable | focal | zone 3 | | diffuse | zone 3 | mixed | mixed，mainly macrovesicular |
| 5 | 15 | 30 | stable | diffuse | zone 3 | | focal | extend to zone 2 and the periportal area | macrovesicular | Mixed |
| 6 | 10 | 30 | moderate progression | diffuse | zone 3 | | focal | zone 3 | mixed | mixed |
| 7 | 10 | 35 | moderate progression | focal | zone 3 | | focal | zone 3 | macrovesicular | mixed |
| 8 | 30 | 50 | moderate progression | focal | extend to zone 2 and the periportal area | | mixed | extend to zone 2 and the periportal area | mixed | mixed |

**Supplementary Table 3. Change in hepatic steatosis among AIH patients who underwent CAP measurements both before and after treatment (CAP cut-off = 248).**

|  | **With Hepatic Steatosis After Treatment** | **Without Hepatic Steatosis After Treatment** |
| --- | --- | --- |
| **With Hepatic Steatosis Before Treatment** | 6 | 2 |
| **Without Hepatic Steatosis Before Treatment** | 10 | 29 |

**Supplementary Table 4. Change in hepatic steatosis among AIH patients who underwent CAP measurements both before and after treatment(CAP cut-off = 274).**

|  | **With Hepatic Steatosis After Treatment** | **Without Hepatic Steatosis After Treatment** |
| --- | --- | --- |
| **With Hepatic Steatosis Before Treatment** | 3 | 2 |
| **Without Hepatic Steatosis Before Treatment** | 11 | 31 |

**Supplementary Table 5. Clinical characteristics of AIH patients who underwent liver ultrasound examinations (n=47)**

| **Variables** | **New-onset Hepatic Steatosis (n = 12)** | **Stable, Regressed, or Without Hepatic Steatosis (n = 35)** | ***P*** |
| --- | --- | --- | --- |
|  |  |  |  |
| **Female(%)** | 12 (100.00) | 27 (77.14) | 0.170 |
| **Age at disease onset(years)** | 50.50 ± 13.27 | 55.37 ± 11.50 | 0.230 |
| **BMI** | 22.28 ± 1.65 | 22.51 ± 3.00 | 0.801 |
| **Initial dose of glucocorticoids(mg)** | 24.00 (23.00, 30.50) | 24.00 (16.00, 27.00) | 0.375 |
| **CBR at 6 month（%）** | 4 (33.33) | 22 (62.86) | 0.076 |
| **ANA(%)** | 6 (50.00) | 25 (71.43) | 0.318 |
| **SMA(%)** | 1 (8.33) | 2 (5.71) | 1.000 |
| **Laboratory test at diagnosis** | | | |
| **RBC（×10^12^/L)** | 3.81 ± 0.48 | 3.88 ± 0.61 | 0.712 |
| **Hb(g/L)** | 119.08 ± 14.78 | 123.51 ± 17.37 | 0.434 |
| **WBC（×10^9^/L)** | 5.66 (3.04, 10.63) | 5.10 (3.87, 9.43) | 0.895 |
| **PLT（×10^9^/L)** | 179.50 (109.50, 206.00) | 127.00 (97.00, 169.50) | 0.105 |
| **ALB(g/L)** | 37.49 ± 4.13 | 36.49 ± 4.49 | 0.498 |
| **GLO(g/L)** | 33.30 (27.77, 37.03) | 29.10 (26.30, 34.90) | 0.414 |
| **ALT(U/L)** | 257.70 (150.08, 407.48) | 122.90 (53.70, 223.70) | **0.014** |
| **AST(U/L)** | 220.80 (184.93, 432.68) | 167.60 (54.50, 284.20) | 0.116 |
| **γ-GT(U/L)** | 147.90 (83.70, 200.75) | 102.90 (58.50, 180.95) | 0.472 |
| **ALP(U/L)** | 91.55 (88.88, 122.90) | 127.10 (100.90, 164.50) | 0.107 |
| **IgG(g/L)** | 16.43 ± 4.38 | 16.96 ± 5.01 | 0.745 |
| **FBG(mmol/L)** | 4.92 (4.21, 5.44) | 4.82 (4.46, 5.44) | 0.990 |
| **Cholesterol(mmol/L)** | 3.79 ± 1.14 | 3.73 ± 1.28 | 0.882 |
| **Triglycerides(mmol/L)** | 1.46 (0.86, 1.82) | 1.26 (0.88, 1.78) | 0.864 |
| **High-density lipoprotein(mmol/L)** | 1.44 (0.91, 1.82) | 0.97 (0.68, 1.74) | 0.442 |
| **Low-density lipoprotein(mmol/L)** | 1.98 ± 0.74 | 1.90 ± 0.85 | 0.790 |
| **Laboratory test after six-month immunosuppressive therapy** | | | |
| **ALT(U/L)** | 23.20 (17.73, 44.62) | 26.10 (16.20, 30.75) | 0.903 |
| **AST(U/L)** | 27.50 (22.32, 43.62) | 27.20 (20.05, 40.40) | 0.373 |
| **γ-GT(U/L)** | 28.70 (18.55, 43.50) | 24.00 (16.45, 43.66) | 0.874 |
| **ALP(U/L)** | 64.15 (54.02, 70.45) | 69.60 (60.10, 84.50) | 0.246 |
| **FBG(mmol/L)** | 5.66 ± 0.91 | 5.28 ± 0.88 | 0.204 |
| **Cholesterol(mmol/L)** | 5.20 (4.67, 6.51) | 4.73 (3.83, 5.15) | 0.081 |
| **Triglycerides(mmol/L)** | 1.51 (0.99, 2.89) | 0.99 (0.83, 1.56) | 0.075 |
| **High-density lipoprotein(mmol/L)** | 1.62 (1.33, 1.71) | 1.52 (1.29, 1.94) | 0.723 |
| **Low-density lipoprotein(mmol/L)** | 2.85 (2.39, 4.48) | 2.65 (2.04, 3.30) | 0.335 |
| **IgG(g/L)** | 14.55 (10.33, 15.72) | 12.00 (10.55, 13.90) | 0.442 |

**Supplementary Table 6. Change in hepatic steatosis among AIH patients who underwent liver ultrasound examinations (n = 47).**

|  | **With Hepatic Steatosis After Treatment** | **Without Hepatic Steatosis After Treatment** |
| --- | --- | --- |
| **With Hepatic Steatosis Before Treatment** | 8 | 2 |
| **Without Hepatic Steatosis Before Treatment** | 12 | 25 |

**Supplementary Table 7.Predictors of new-onset hepatic steatosis after treatment in AIH patients who underwent liver ultrasound examinations before and after treatment**

| **Variables** | **Univariate analysis** | |  | **Multivariate analysis** | |
| --- | --- | --- | --- | --- | --- |
|  | **OR(95%CI)** | **P value** |  | **OR(95%CI)** | **P value** |
| **ALT at diagnosis** | 1.00 (1.00 ~ 1.00) | 0.073 |  | 1.01 (1.01 ~ 1.01) | **0.023** |
| **ALP at diagnosis** | 0.99 (0.97 ~ 1.00) | 0.099 |  | 0.96 (0.92 ~ 0.99) | **0.021** |
| **TC after six months of treatment** | 1.89 (1.07 ~ 3.33) | **0.028** |  |  |  |
| **TG after six months of treatment** | 2.17 (0.99 ~ 4.78) | 0.054 |  | 1.90 (0.94 ~ 3.83) | 0.072 |

**Supplementary Table 8. Internal consistency analysis among patients with all three paired assessment modalities**

| **Comparison** | **Cohen's Kappa**  **(before treatment)** | **Cohen's Kappa**  **(after treatment)** |
| --- | --- | --- |
| **Liver biopsy vs CAP** | 0.82 | 0.92 |
| **Liver biopsy vs Liver ultrasound examinations** | 0.72 | 0.77 |
| **CAP vs Liver ultrasound examinations** | 0.91 | 0.85 |

**Supplementary Table 9. Component analysis of new-onset and progressive hepatic steatosis**

| **Assessment modality** |  | **Composite endpoint** | **New-onset steatosis** | **Progression of pre-existing steatosis** |
| --- | --- | --- | --- | --- |
| **Liver biopsy** |  | 18 | 13 | 5 |
| **CAP** |  | 13 | 10 | 3 |
| **Ultrasound** |  | 12 | 12 | NA |
| **Overall** |  | 43 | 35 | 8 |

*Progression was quantified only in the biopsy and CAP subgroups. Ultrasound-defined progression was not assessed because ultrasound was recorded as a binary variable in routine clinical practice.*

**Supplementary Table 10. Univariable and multivariable logistic regression analyses for new-onset hepatic steatosis among patients without hepatic steatosis at baseline**

| **Variables** | **Univariate analysis** | |  | **Multivariate analysis** | |
| --- | --- | --- | --- | --- | --- |
|  | **OR(95%CI)** | ***P* value** |  | **OR(95%CI)** | ***P* value** |
| **Initial dose of glucocorticoids** | 1.07 (1.01 ~ 1.14) | **0.033** |  |  |  |
| **RBC at diagnosis** | 2.79 (1.29 ~ 6.05) | **0.009** |  | 2.43 (1.02 ~ 5.81) | **0.046** |
| **PLT at diagnosis** | 1.01 (1.01 ~ 1.01) | **0.020** |  |  |  |
| **ALB at diagnosis** | 1.09 (1.01 ~ 1.18) | **0.049** |  |  |  |
| **ALP after six months of treatment** | 0.98 (0.97 ~ 0.99) | **0.049** |  | 0.98 (0.97 ~ 1.00) | 0.071 |
| **TC after six months of treatment** | 1.58 (1.17 ~ 2.13) | **0.003** |  |  |  |
| **TG after six months of treatment** | 2.57 (1.59 ~ 4.14) | **<0.001** |  | 2.78 (1.61 ~ 4.78) | **<0.001** |

**Supplementary Table 11. Progression of pre-existing steatosis was assessed only in patients with baseline steatosis evaluated by liver biopsy or CAP**

| **Variables** | **Progression** | **Non-progression** | ***P* value** |
| --- | --- | --- | --- |
| **BMI** | 26.09（24.74, 26.56） | 23.54（21.83, 24.95） | **0.037** |
| **RBC at diagnosis** | 4.48（4.31, 4.87） | 4.12（3.81, 4.47） | 0.061 |
| **ALB at diagnosis** | 41.05（39.68, 41.55） | 37.70（36.10, 41.60） | 0.098 |
| **TC after six months of treatment** | 6.33（5.91, 6.60） | 5.09（4.80, 5.91） | 0.071 |
| **TG after six months of treatment** | 2.08（1.28, 2.42） | 1.44（1.04, 1.80） | 0.184 |
| **LDL-C after six months of treatment** | 4.29（3.55, 4.74） | 2.79（2.26, 3.27） | **0.017** |

**Supplementary Table 12. Sensitivity analysis including baseline BMI and baseline TG in the multivariable model**

| **Variables** | **Univariate analysis** | |  | **Multivariate analysis** | |
| --- | --- | --- | --- | --- | --- |
|  | **OR(95%CI)** | ***P* value** |  | **OR(95%CI)** | ***P* value** |
| **Female** | 2.17 (0.78 ~ 6.08) | 0.140 |  | 4.26 (1.18 ~ 15.38) | **0.027** |
| **RBC at diagnosis** | 2.91 (1.46 ~ 5.79) | **0.002** |  | 4.33 (1.70 ~ 11.01) | **0.002** |
| **TG at diagnosis** | 1.14 (0.77 ~ 1.69) | 0.502 |  | 0.58 (0.30 ~ 1.14) | 0.116 |
| **BMI at diagnosis** | 1.09 (0.98 ~ 1.22) | 0.121 |  | 1.07 (0.94 ~ 1.22) | 0.288 |
| **TG after six months of treatment** | 2.48 (1.62 ~ 3.79) | **<0.001** |  | 2.56 (1.61 ~ 4.08) | **<0.001** |

**Supplementary Table 13. Exploratory interaction analysis between 6-month triglycerides and BMI for new-onset or progressive hepatic steatosis**

| **Variable** | **OR (95%CI)** | ***P* value** |
| --- | --- | --- |
| **Female** | 4.41 (1.19 ~ 16.28) | 0.026 |
| **RBC at diagnosis** | 3.61 (1.47 ~ 8.84) | 0.005 |
| **TG after six months of treatment, centered** | 2.48 (1.59 ~ 3.87) | <0.001 |
| **BMI, centered** | 1.04 (0.91 ~ 1.18) | 0.600 |
| **TG × BMI interaction** | 1.19 (0.99 ~ 1.42) | 0.065 |

**Supplementary Table 14. Exploratory interaction analysis between 6-month triglycerides and baseline CAP in the paired CAP subgroup**

| **Variable** | **OR (95%CI)** | ***P* value** |
| --- | --- | --- |
| **BMI, centered** | 1.33 (0.99 ~ 1.79) | 0.058 |
| **6-month TG, centered** | 2.69 (1.10 ~ 6.58) | 0.030 |
| **Baseline CAP, centered, per 10 dB/m** | 0.98 (0.84 ~ 1.16) | 0.847 |
| **6-month TG × baseline CAP interaction** | 1.14 (0.93 ~ 1.40) | 0.211 |
